# Costs of Drug Development and Research and Development Intensity in the US, 2000-2018

**DOI:** 10.1001/jamanetworkopen.2024.15445

**Published:** 2024-06-28

**Authors:** Aylin Sertkaya, Trinidad Beleche, Amber Jessup, Benjamin D. Sommers

**Affiliations:** 1 Eastern Research Group, Inc, Lexington, Massachusetts; 2 Office of the Assistant Secretary for Planning and Evaluation, Office of Science and Data Policy, US Department of Health and Human Services, Washington, DC; 3 Now with Office of Inspector General, US Department of Health and Human Services, Washington, DC; 4 US Department of Health and Human Services, Washington, DC; 5 Now with Department of Health Policy and Management, Harvard T.H. Chan School of Public Health, Boston, Massachusetts

## Abstract

**Importance:**

Understanding the cost of drug development can help inform the development of policies to reduce costs, encourage innovation, and improve patient access to drugs.

**Objective:**

To estimate the cost of drug development by therapeutic class and trends in pharmaceutical research and development (R&D) intensity over time.

**Design, Setting, and Participants:**

In this economic evaluation study, an analytical model of drug development constructed using public and proprietary sources that collectively cover data from 2000 to 2018 was used to estimate the cost of bringing a drug to market, overall and for specific therapeutic classes. The analysis for the study was completed in October 2020.

**Main Outcomes and Measures:**

Three measures of development cost from nonclinical through postmarketing stages were estimated: mean out-of-pocket cost or cash outlay, mean expected cost, and mean expected capitalized cost. Pharmaceutical R&D intensity, defined as the ratio of R&D spending to total sales, from 2008 to 2019, based on the time frame for available data, was also analyzed.

**Results:**

The estimated mean cost of developing a new drug was approximately $172.7 million (2018 dollars) (range, $72.5 million for genitourinary to $297.2 million for pain and anesthesia), inclusive of postmarketing studies. The cost increased to $515.8 million when cost of failures was included. When the costs of failures and capital were included, the mean expected capitalized cost of drug development increased to $879.3 million (range, $378.7 million for anti-infectives to $1756.2 million for pain and anesthesia); results varied widely by therapeutic class. The pharmaceutical industry as a whole experienced a decline of 15.6% in sales but increased R&D intensity from 11.9% to 17.7% from 2008 to 2019. By contrast, R&D intensity of large pharmaceutical companies increased from 16.6% to 19.3%, whereas sales increased by 10.0% (from $380.0 to $418.0 billion) over the same 2008 to 2019 period, even though the cost of drug development remained relatively stable or may have even decreased.

**Conclusions and Relevance:**

In this economic evaluation of new drug development costs, even though the cost of drug development appears to have remained stable, R&D intensity of large pharmaceutical companies remained relatively unchanged, despite substantial growth in revenues during this period. These findings can inform the design of drug-related policies and their potential impacts on innovation and competition.

## Key Points

**Question:**

What is the mean cost of drug development for the US market, and how has research and development (R&D) intensity changed over time?

**Findings:**

This economic evaluation study used data from public and proprietary sources to estimate the mean cost of developing a new drug from 2000 to 2018, which was $172.7 million (2018 dollars) but increased to $515.8 million when cost of failures was included and to $879.3 million when both drug development failure and capital costs were included. The ratio of R&D spending to total sales increased from 11.9% to 17.7% from 2008 to 2019.

**Meaning:**

In this study, the cost of drug development increased by a factor of 5 when accounting for costs of capital and failures; these findings can help inform the development of policies to reduce costs, encourage innovation, and improve patient access to drugs.

## Teaser

This economic evaluation estimates the cost of drug development by therapeutic class and trends in pharmaceutical research and development intensity in the US from 2000 to 2018.

## Introduction

High drug prices increase the likelihood of medication nonadherence and are an ongoing public health concern in the US. US drug prices are 2 to 3 times higher than those in other countries^1,2,3^ and are even higher for some critical medications such as insulin.^4^ Moreover, drug price increases in recent years have far outpaced the rate of inflation.^5,6^

Efforts to curb high drug pricing have sometimes been criticized for their potential to stunt innovation. Some argue that high drug prices are needed to recoup investments in research and development (R&D). Several recent studies^7,8^ project that even modest decreases in drug prices could lead to reductions in R&D, which could result in fewer new drugs coming to market. Some studies,^9^ by contrast, have found no association between R&D investment and the price of drugs sold in the US.

Manufacturers contend that the length and cost of developing new drugs are the primary contributing factors to high drug prices. Studies have estimated that the R&D cost for a new drug ranges from $314 million to $4.46 billion, depending on the therapeutic area, data, and modeling assumptions.^10,11,12,13,14,15,16,17,18,19,20^ Others argue that high drug prices are due to monopolistic behavior and excess profits for large pharmaceutical companies compared with other industries.^21,22,23,24,25^

In this study, we developed a transparent analytical model to better understand the scale and underlying factors of R&D cost associated with bringing a new drug to the US market. Our model was developed using per-patient costs estimated from actual negotiated clinical trial contracts data from 2000 to 2018; our approach is in contrast with most previous studies, which have used manufacturers’ self-reported aggregate data on development costs. We also examined trends in R&D intensity and total sales. The extent to which R&D costs are underlying overall drug development costs is a key factor in understanding the potential effect of changes in drug pricing on new product development. Combined, these findings can inform the design of drug-related policies and their potential impacts on innovation.

## Methods

This economic evaluation study used existing data sources without any personal identifiable information and, hence, was exempt from institutional review board or ethics committee review per the Common Rule (45 CFR §46). This study followed the relevant portions of the Consolidated Health Economic Evaluation Reporting Standards (CHEERS) reporting guideline for economic evaluations.

### Data and Methods for Cost of Drug Development Analysis

We used several public and proprietary data sources and published estimates to estimate our model parameters on per-patient costs, number of trials, number of patients, phase transition probabilities, and duration associated with the various stages of clinical trial development. These data sources included ClinicalTrials.gov and custom tabulations from the US Food and Drug Administration (FDA) internal drug databases, Medidata Solutions, and IQVIA’s GrantPlan databases that contain cost information on thousands of actual negotiated contracts for clinical trials funded by pharmaceutical companies. These databases included information that spanned from 2000 to 2018.

**Figure 1.**
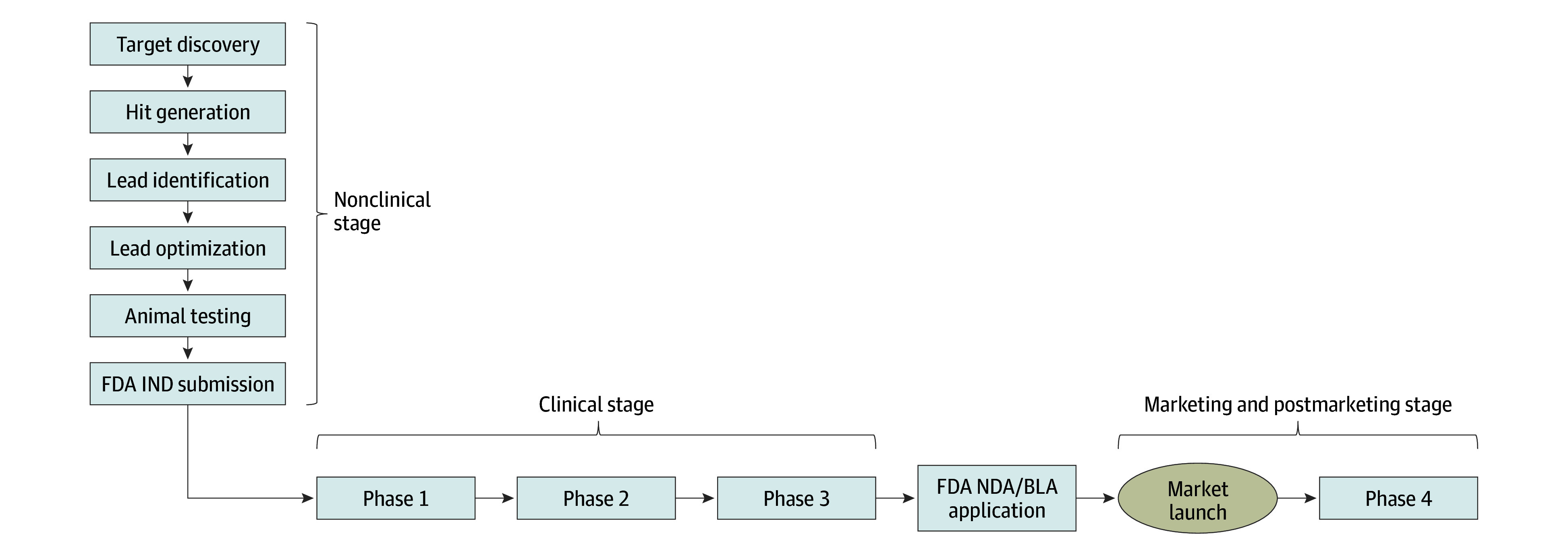
Stages of Drug Development BLA indicates Biologic License Application; FDA, US Food and Drug Administration; IND, Investigational New Drug; NDA, New Drug Application.

Figure 1 presents a stylized model of drug development by stages: nonclinical, clinical (phase 1 to phase 3), FDA review, and postmarketing (phase 4). On the basis of the framework in Figure 1, we estimated 3 measures of drug development cost using the approach by DiMasi et al.^17^ The first measure, cost, represents the mean cash outlay paid for a single approved drug from the nonclinical stage through postmarketing. The second measure represents the expected cost, which includes the cost for successful drugs as well as expenditures on drugs that fail at some stage of the process. The drug development process involves risks, and not all drugs make it to market. To incorporate the varying probabilities of failure at each stage of the process, we calculated the expected cost of each drug development stage by dividing the cost estimated for that stage by the mean aggregate probability of the drug successfully making it to market from that stage. Our third measure represents the expected capitalized cost, which accounts for duration of the development process and the associated opportunity cost of capital. The expected capitalized cost was calculated by applying continuous compounding, assuming that the expected cost is distributed uniformly over the duration of that stage, as described in DiMasi et al,^17^ using an inflation-adjusted cost of capital appropriate for the pharmaceutical industry. Finally, we calculated total cost, expected cost, and expected capitalized cost per drug by summing the corresponding costs for each stage of the drug development process. For example, we calculated the expected cost of phase 1 by dividing the cost of conducting a phase 1 study by the mean aggregate probability of a drug successfully making it to market from phase 1. We then calculated the expected capitalized cost of phase 1 by applying continuous compounding, assuming that this cost is distributed uniformly over the duration of phase 1 (see the eAppendix in Supplement 1).

For each development stage and across 13 therapeutic areas, we estimated 7 parameters: phase duration; duration from start of one phase to the start of next phase, referred to as start to start (because phases tend to overlap); per-patient cost; number of patients enrolled per trial; mean number of trials conducted in support of a new drug or biologics license application under a given investigational drug application number; phase transition success probabilities; and inflation-adjusted cost of capital. Our clinical costs were estimated as follows: in brief, phase duration represents the time it takes to complete a given phase of drug development. Start to start time represents the elapsed time in months between the start of one development stage (eg, phase 2) supporting a new drug or biologics license application and the start of the next development stage (eg, phase 3). The per-patient cost represents the mean cost that a sponsor incurs per-patient in a clinical study in 2018 dollars inclusive of all overhead costs, which we estimated using 3 different proprietary databases on clinical trial costs (eTable 1 in Supplement 1). The number of patients enrolled represents the mean number of patients enrolled for a given clinical study (phase 1, 2, 3, or phase 4) estimated as the weighted mean number of patients reported for trials in ClinicalTrials.gov, Medidata, and FDA internal databases where the weights were the number of trials (eTable 2 in Supplement 1). The mean number of trials represents the number of trials a sponsor conducts under an investigational new drug number and is based on a custom tabulation from FDA internal databases (eTable 3 in Supplement 1). The transition success probabilities reflect the probability of a sponsor successfully moving from one stage of drug development to the next and are estimated using published values in peer-reviewed and gray literature (eTable 4 in Supplement 1). For example, if there are 100 drugs at the phase 1 and only 30 are successful and subsequently begin phase 2 studies, then the transition success probability from phase 1 to phase 2 is 30%. Finally, the real cost of capital represents the rate of inflation-adjusted return that the sponsor would otherwise be able to earn at the same risk level as the investment in the drug candidate that has been selected. This value varies greatly by sponsor-specific factors (eg, product portfolio and size of company) and other exogenous factors (eg, economic and regulatory climate for drug development). The estimated value for the biopharmaceutical sector ranges from 8.1% to as high as 14.5%. We used 11.0% as the real cost of capital (eTable 5 in Supplement 1).

The nonclinical stage cost was then estimated according to the ratio of preclinical costs to total clinical costs in DiMasi et al,^17^ which was 44.6% for approved drugs; we applied this percentage to our own sample’s estimated cost within each drug class for approved drugs to derive a per-drug candidate preclinical cost (and assumed it was the same, whether or not a drug candidate ultimately went on to receive approval). The eAppendix in Supplement 1 contains additional information regarding how and why we used the various sources to estimate each parameter.

### Data and Methods for Trend Analysis of Pharmaceutical Industry R&D Intensity and Sales

To assess R&D intensity and sales over time, we used the National Science Foundation Business Enterprise Research and Development Survey (BERD) from 2008 to 2019, the most recent years for which the data were consistently available. The BERD data are collected annually from a probability sample of for-profit companies with 10 or more employees and a US presence. The BERD data are reported using a 5-digit North American Industry Classification System code. Although aggregate data on worldwide R&D spending and total sales are available by company size, a breakdown by size is not publicly available by NAICS codes. We used worldwide R&D spending and total sales reported for North American Industry Classification System codes 32541, 32542, and 32543, which comprise the pharmaceutical industry. Sales refers to the total product sales a company generates net of discounts, rebates, allowances, returns, and commissions and excludes royalty payments, interest, and dividends.

We supplemented BERD data with worldwide R&D expenditures and sales for 2008 to 2019 as reported by Pharmaceutical Research and Manufacturers of America (PhRMA) 2020 Annual Survey.^26^ The 2020 PhRMA Annual Survey includes R&D expenditures and sales from its member companies only, which are primarily large manufacturers. To be eligible for PhRMA membership, a company must have an average global R&D to global sales ratio of 10 or greater and 3-year average global R&D spending of at least $200 million per year.^27^

We examined trends in R&D spending and sales (net of discounts and rebates), which were adjusted to real 2018 US dollars using the Medical Care Consumer Price Index. R&D spending denotes costs a company incurs as it works to improve, design, and create new products, services, technologies, or processes. These expenses are considered direct costs and are separate from other business expenses. R&D intensity is a measure used to assess innovative activity and is defined as the ratio of R&D spending to sales.

### Statistical Analysis

The analysis for the study was completed in October 2020. Given the nature of the data used in the modeling, we were not able to assess statistical significance. We used nonparametric bootstrapped resampling with replacement method (10 000 iterations) to calculate 95% CIs for the estimates without making any distributional assumptions about the underlying data. The bootstrapped parameters included phase duration, number of patients enrolled per trial, per-patient cost, phase transition success probability, and cost of capital. The values for each were sampled with replacement from eTables 1, 2, 4, 5, and 6 in Supplement 1. We used @Risk software version 8.0.0 Industrial Edition (Palisade Company) to calculate and organize the data.

## Results

### Estimated Mean Cost of Drug Development Overall and by Therapeutic Area

Based on the model parameters (Table 1), the estimated mean cost of developing a new drug was approximately $172.7 million (95% CI, $132.5-$197.9 million; range, $72.5 million for genitourinary to $297.2 million for pain and anesthesia) (Table 2). If the cost of failures is considered, this figure, which we label expected cost, increases to $515.8 million (95% CI, $327.0-$773.2 million). When the costs of capital are included, the expected capitalized cost becomes $879.3 million (95% CI, $416.9-$1307.3 million). These costs vary widely by therapeutic area. At one end of the spectrum are anti-infective drugs, which cost approximately one-third of the estimated mean (inclusive of cost of capital and cost of failures) at $378.7 million (95% CI, $244.6-$556.1 million), and at the other end are pain and anesthesia drugs, which were more than twice as costly to develop at $1756.2 million (95% CI, $648.5-$3171.5 million). The next most expensive drugs to develop included oncology at $1209.2 million (95% CI, $624.6-$2388.7 million) followed by ophthalmology drugs at $1191.6 million (95% CI, $496.3-$1910.8 million).

**Table 1.** Drug Development Cost Model Parameters and Assumptions by Therapeutic Area and Phase^a^

Parameter and phase	Anti-infective	Cardio-vascular	Central nervous system	Derma-tology	Endocrine	Gastro-intestinal	Genito-urinary system	Hema-tology	Oncology	Respira-tory system	Ophthal-mology	Pain and anesthesia	Immuno-modulation	All
Phase durations, mo														
Nonclinical	31.2	31.2	31.2	31.2	31.2	31.2	31.2	31.2	31.2	31.2	31.2	31.2	31.2	31.2
Phase 1	21.5	14.1	19.5	27.8	12.9	27.8	12.4	23.8	31.9	17.6	17.9	27.8	13.1	27.8
Phase 2	28.0	38.0	40.1	34.0	29.3	34.0	25.8	35.0	40.3	37.1	27.0	34.0	33.1	34.0
Phase 3	32.8	42.0	45.3	38.0	35.6	38.0	33.0	58.3	57.7	42.2	33.7	38.0	36.3	38.0
FDA review	14.8	19.1	21.0	12.2	18.8	17.9	18.3	15.3	9.6	18.9	11.9	31.7	16.8	16.2
Phase 4	38.7	38.5	35.0	36.6	34.0	36.6	29.9	36.6	45.7	36.6	30.7	36.6	39.6	36.6
Start to start, mo														
Nonclinical to phase 1	31.2	31.2	31.2	31.2	31.2	31.2	31.2	31.2	31.2	31.2	31.2	31.2	31.2	31.2
Phase 1 to phase 2	12.9	8.5	11.7	16.6	7.7	16.6	7.4	14.2	19.1	10.5	10.7	16.6	7.9	16.6
Phase 2 to phase 3	22.4	30.4	32.1	27.2	23.4	27.2	20.6	28.0	32.2	29.7	21.6	27.2	26.5	27.2
Phase 3 to FDA review	22.3	28.6	30.8	25.9	24.2	25.9	22.4	39.7	39.3	28.7	22.9	25.9	24.7	25.9
FDA review to approval	14.8	19.1	21.0	12.2	18.8	17.9	18.3	15.3	9.6	18.9	11.9	31.7	16.8	16.2
Per-patient cost (2018), $														
Nonclinical	NA	NA	NA	NA	NA	NA	NA	NA	NA	NA	NA	NA	NA	NA
Phase 1	19 399	59 456	87 390	35 450	85 463	61 848	53 770	349 363	103 344	44 330	50 999	90 370	63 471	81 338
Phase 2	59 289	41 323	48 767	66 661	51 556	63 590	45 781	100 554	78 753	43 563	48 438	77 726	47 897	58 618
Phase 3	30 001	33 084	39 612	48 587	48 753	47 656	38 930	118 473	93 145	46 764	79 933	60 751	54 909	53 180
FDA review	NA	NA	NA	NA	NA	NA	NA	NA	NA	NA	NA	NA	NA	NA
Phase 4	13 814	33 915	34 956	33 102	56 824	52 746	16 699	41 958	23 515	18 987	24 022	41 573	30 246	35 190
No. of patients enrolled per trial														
Nonclinical	NA	NA	NA	NA	NA	NA	NA	NA	NA	NA	NA	NA	NA	NA
Phase 1	69	42	44	106	38	38	50	31	58	49	121	36	55	51
Phase 2	243	189	243	133	225	292	323	134	137	203	299	270	323	235
Phase 3	575	1151	529	568	414	496	546	233	293	516	876	1209	309	630
FDA review	NA	NA	NA	NA	NA	NA	NA	NA	NA	NA	NA	NA	NA	NA
Phase 4	1430	508	356	850	482	1344	410	411	261	1159	413	280	383	708
Mean No. of trials														
Nonclinical	NA	NA	NA	NA	NA	NA	NA	NA	NA	NA	NA	NA	NA	NA
Phase 1	2	2	2	2	2	2	2	2	1	1	1	2	2	2
Phase 2	2	1	1	2	2	1	1	2	1	2	2	2	2	2
Phase 3	2	2	3	3	4	2	1	2	2	4	2	3	3	3
FDA review	NA	NA	NA	NA	NA	NA	NA	NA	NA	NA	NA	NA	NA	NA
Phase 4	2	2	2	1	2	2	1	2	1	2	2	1	2	2
Transition success probabilities, %														
Nonclinical to phase 1	68.0	68.0	68.0	68.0	68.0	68.0	68.0	68.0	68.0	68.0	68.0	68.0	68.0	68.0
Phase 1 to phase 2	65.9	65.0	61.5	60.2	68.0	71.6	62.9	73.3	61.5	68.5	86.0	60.2	69.9	60.2
Phase 2 to phase 3	49.6	37.1	33.1	35.9	46.3	35.3	44.9	56.6	26.8	27.4	52.7	35.9	40.1	35.9
Phase 3 to FDA review	74.1	57.6	55.9	65.5	63.9	55.3	71.4	75.0	42.7	75.6	58.3	65.5	65.4	65.5
FDA review to approval	94.4	75.5	87.0	88.3	81.3	86.2	85.7	84.0	85.5	89.5	77.5	88.3	95.3	88.3
Out-of-pocket estimates (millions), $														
Nonclinical	9.4	10.1	8.7	9.3	14.2	11.4	7.7	17.9	7.0	9.0	32.0	22.2	11.6	11.8
Phase 1	2.8	4.2	6.8	6.5	6.8	4.3	4.2	17.3	8.1	3.2	7.6	6.1	6.8	7.1
Phase 2	22.3	11.2	16.1	14.9	19.4	26.2	19.8	22.1	14.5	13.7	22.8	34.6	24.3	21.0
Phase 3	41.6	91.2	59.4	70.6	85.8	58.1	31.3	61.9	37.7	90.5	173.0	214.4	52.4	89.3
FDA review	2.6	2.6	2.6	2.6	2.6	2.6	2.6	2.6	2.6	2.6	2.6	2.6	2.6	2.6

Abbreviations: FDA, Food and Drug Administration; NA, not applicable.

^a^ The total percentage cost of capital is 11%.

**Table 2.** Mean Drug Development Cost Across All Therapeutic Areas^a^

Therapeutic area	Overall probability of success, % (95% CI)^b^	Cost, millions of 2018 $ (95% CI)		
		Cost^c^	Expected cost^d^	Expected capitalized cost^e^
Anti-infective	15.5 (10.9-21.7)	109.4 (65.3-186.7)	251.0 (166.7-360.3)	378.7 (244.6-556.1)
Cardiovascular	7.1 (3.6-15.3)	153.5 (73.7-593.3)	519.3 (273.9-2168.1)	890.3 (445.0-3807.9)
Central nervous system	6.7 (4.0-12.9)	113.0 (84.9-132.0)	463.7 (302.5-714.3)	894.6 (526.1-1550.3)
Dermatology	8.5 (8.5-8.5)	143.8 (60.0-253.0)	419.1 (221.5-688.9)	683.1 (349.5-1252.2)
Endocrine	11.1 (6.2-17.7)	181.2 (139.2-226.8)	492.0 (359.4-728.4)	780.0 (541.0-1243.2)
Gastrointestinal	8.2 (6.4-10.3)	216.7 (101.4-363.6)	591.1 (285.2-898.3)	963.5 (451.5-1564.5)
Genitourinary system	11.8 (7.8-16.3)	72.5 (52.7-83.7)	244.0 (152.4-345.8)	394.9 (228.9-626.7)
Hematology	17.8 (17.8-17.8)	152.9 (82.5-230.7)	381.9 (214.3-578.0)	720.4 (348.2-1295.1)
Oncology	4.1 (2.2-8.6)	84.7 (65.5-121.6)	595.5 (344.3-1041.0)	1209.2 (624.6-2388.7)
Respiratory system	8.6 (5.3-11.7)	167.8 (94.0-332.2)	409.3 (246.3-685.5)	686.4 (399.5-1164.1)
Ophthalmology	13.9 (11.6-16.2)	256.2 (101.2-381.8)	787.6 (333.4-1179.7)	1191.6 (496.3-1910.8)
Pain and anesthesia	8.5 (8.5-8.5)	297.2 (82.8-515.9)	887.9 (325.3-1476.9)	1756.2 (648.5-3171.5)
Immunomodulation	11.9 (7.5-18)	119.3 (74.0-180.1)	363.7 (201.7-586.2)	605.8 (306.1-1041.9)
Overall	8.5 (3.9-16.1)	172.7 (132.5-197.9)	515.8 (327.0-773.2)	879.3 (416.9-1307.3)

^a^ Some drugs were approved on the basis of phase 2 trials only. Therefore, the estimated total costs presented in this study are not representative of those drug development programs.

^b^ Represents the probability of successfully making it to approval from the nonclinical stage.

^c^ Represents the cash outlay not adjusted for the cost of capital or failures.

^d^ Represents research and development cost after adjusting for the cost of failures computed as the total cash outlay divided by the aggregate transition success probability; includes cost of failures but not the cost of capital.

^e^ Represents costs inclusive of failure and capital costs.

Table 3 shows costs by stage. Across all therapeutic areas, the nonclinical stage accounted for 6.8% (95% CI, 3.7%-9.1%) of mean development cost. This share varied from 5.3% (95% CI, 2.2%-9.4%) for gastrointestinal to as high as 12.5% (95% CI, 9.3%-15.4%) for ophthalmology drug development. The share of the nonclinical stage increased to 27.0% (95% CI, 22.1%-28.1%) of the total when failure cost was accounted for, and to 40.2% (95% CI, 35.2%-44.6%) when both failure and capital costs were considered. Across the therapeutic areas, the expected nonclinical stage cost as share of total cost ranged from 23.7% (95% CI 15.6%-28.1%) for gastrointestinal to 29.4% (95% CI, 26.0%-30.2%) for pain and anesthesia drug development. The expected capitalized nonclinical stage cost as share of total ranged from 35.9% (95% CI, 28.9%-42.2%) for anti-infective to 43.5% (95% CI, 37.3%-49.6%) for pain and anesthesia drug development. See eTable 7 in Supplement 1 for sensitivity analysis on nonclinical costs.

**Table 3.** Nonclinical, Clinical, FDA Review, and Postapproval Costs as Percentage of Mean, Mean Expected, and Mean Expected Capitalized Total Cost^a^

Therapeutic area and type of cost	Percentage of total cost (95% CI)			
	Nonclinical	Clinical	FDA review	Phase 4
Anti-infective				
Cost	8.6 (4.9-12.5)	61.0 (37.6-81.2)	2.4 (1.4-4.0)	28.0 (5.9-55.2)
Expected cost	24.2 (18.4-27.7)	54.2 (41.2-62.1)	9.4 (5.9-15.3)	12.2 (2.3-31.4)
Expected capitalized cost	35.9 (28.9-42.2)	50.6 (42.7-55.1)	6.7 (3.8-11.7)	6.8 (1.2-19.9)
Cardiovascular				
Cost	6.6 (3.9-12.3)	69.4 (43.1-92.4)	1.7 (0.4-3.5)	22.3 (2.2-50.1)
Expected cost	27.4 (22.9-30.3)	61.5 (51.5-68.0)	4.5 (1.0-9.2)	6.6 (0.6-18.4)
Expected capitalized cost	40.8 (34.4-49.7)	53 (47.3-57.4)	2.9 (0.6-6.4)	3.2 (0.3-9.9)
Central nervous system				
Cost	7.7 (5.3-10.6)	72.8 (65.1-84.5)	2.3 (2.0-3.0)	17.2 (4.4-25.2)
Expected cost	28.0 (26-29.3)	62.8 (58.5-65.7)	5.1 (3.0-8.8)	4.2 (0.9-7.8)
Expected capitalized cost	40.2 (35.8-45.7)	55.0 (50.4-58.5)	2.9 (1.4-5.8)	1.9 (0.4-3.9)
Dermatology				
Cost	6.5 (3.2-12.1)	64.0 (30.7-88.0)	1.8 (1.0-4.3)	27.7 (2.4-64.6)
Expected cost	26.2 (19.0-29.4)	58.7 (42.7-65.9)	5.6 (3.2-11.6)	9.5 (0.6-31.7)
Expected capitalized cost	39.3 (31.8-46.8)	52.1 (42.6-58.6)	3.7 (1.6-8.2)	5.0 (0.3-18.8)
Endocrine				
Cost	7.9 (5.3-11.4)	61.8 (51.0-75.0)	1.4 (1.1-1.9)	28.9 (14.4-41.7)
Expected cost	26.1 (23.1-28.4)	58.5 (51.9-63.8)	4.8 (2.9-7.3)	10.6 (4.1-19.1)
Expected capitalized cost	37.5 (32.9-42.7)	53.4 (48.9-57.3)	3.3 (1.8-5.6)	5.8 (2.0-11.4)
Gastrointestinal				
Cost	5.3 (2.2-9.4)	40.9 (18.3-66.2)	1.2 (0.7-2.6)	52.6 (23.6-78.6)
Expected cost	23.7 (15.6-28.1)	53.1 (34.9-63.0)	4.0 (2.3-8.7)	19.3 (5.9-44.2)
Expected capitalized cost	37.4 (28.2-44.6)	49.9 (38.3-55.7)	2.7 (1.3-6.3)	10.1 (2.6-28.4)
Genitourinary system				
Cost	10.7 (7.7-13.1)	76.3 (70.4-81.6)	3.6 (3.1-4.9)	9.5 (4.4-14.4)
Expected cost	27.0 (24.3-28.4)	60.6 (54.6-63.8)	9.7 (5.7-16.8)	2.8 (1.1-5.0)
Expected capitalized cost	36.2 (32.5-40)	55.8 (51.0-58.2)	6.5 (3.3-12.5)	1.5 (0.6-2.9)
Hematology				
Cost	11.7 (7.9-15.6)	66.2 (46.8-82.0)	1.7 (1.1-3.1)	20.3 (2.3-43.8)
Expected cost	26.4 (21.4-29.0)	59.3 (48.1-65.0)	6.2 (3.5-12.4)	8.1 (0.8-21.1)
Expected capitalized cost	39.5 (34.0-45.8)	53.3 (45.4-57.8)	3.5 (1.6-8.4)	3.7 (0.4-11.3)
Oncology				
Cost	8.3 (5.3-11.1)	71.2 (49.0-83.2)	3.1 (2.1-4.0)	17.5 (4.9-43.0)
Expected cost	28.8 (26.5-29.9)	64.7 (59.4-67.1)	4.0 (2.1-7.4)	2.5 (0.5-9.3)
Expected capitalized cost	41.1 (36.8-46.7)	55.9 (50.9-59.6)	2.0 (0.9-4.5)	1.0 (0.2-4.1)
Respiratory system				
Cost	5.3 (2.3-8.9)	64.0 (29.9-89.9)	1.5 (0.8-2.8)	29.1 (2.0-66.8)
Expected cost	25.4 (16.6-29.3)	56.9 (37.3-65.7)	5.8 (3.2-10.5)	11.9 (0.6-40.9)
Expected capitalized cost	39.1 (29.4-46.2)	51.1 (39.1-56.9)	3.7 (1.9-7.4)	6.0 (0.3-25.8)
Ophthalmology				
Cost	12.5 (9.3-15.4)	79.4 (59.1-86.5)	1.0 (0.7-2.6)	7.1 (1.3-28.8)
Expected cost	29.2 (25.9-30.1)	65.5 (58.1-67.5)	3.0 (1.7-7.6)	2.3 (0.4-9.9)
Expected capitalized cost	41.3 (35.6-46.1)	55.3 (50.6-58.3)	2.1 (1.1-5.6)	1.3 (0.2-6.0)
Pain and anesthesia				
Cost	7.5 (5.8-11.7)	85.8 (57.3-91.4)	0.9 (0.5-3.1)	5.8 (1.3-33.5)
Expected cost	29.4 (26.0-30.2)	66.0 (58.3-67.9)	2.7 (1.4-7.4)	2.0 (0.4-10.6)
Expected capitalized cost	43.5 (37.3-49.6)	54.1 (49.0 -57.9)	1.6 (0.7-4.6)	0.8 (0.2-4.6)
Immunomodulation				
Cost	9.8 (6.2-12.8)	70.0 (54.4-83.1)	2.2 (1.4-3.5)	18.0 (4.9-36.5)
Expected cost	27 (23.1-29.1)	60.6 (51.9-65.2)	6.5 (3.7-12.7)	5.9 (1.2-15.2)
Expected capitalized cost	37.6 (33.1-42.3)	55.2 (49.1-58.8)	4.2 (2.1-9.3)	3.0 (0.6-8.4)
All therapeutic areas				
Cost	6.8 (3.7-9.1)	68.0 (45.8-73.3)	1.5 (1.3-2.0)	23.7 (17.7-47.7)
Expected cost	27.0 (22.1-28.1)	60.5 (49.5-63.1)	4.6 (2.8-8.0)	7.9 (5.1-21.3)
Expected capitalized cost	40.2 (35.2-44.6)	53.0 (48.4-56.9)	2.9 (1.4-5.4)	4.0 (2.4-13.1)

Abbreviation: FDA, US Food and Drug Administration.

^a^ Cost represents the total cash outlay. Expected cost represents the total cash outlay adjusted for cost of failures. Expected capitalized cost represent the total cash outlay adjusted for cost of failures and cost of capital.

The mean cost of the clinical phase per drug candidate was estimated at $117.4 million (data not shown). The clinical stage (phase 1, 2, and 3) accounted for 68.0% (95% CI, 45.8%-73.3%) of total mean costs. The clinical stage cost as a share of total development ranged from 40.9% (95% CI, 18.3%-66.2%) for gastrointestinal drugs to 85.8% (95% CI, 57.3%-91.4%) for pain and anesthesia drugs. The share of clinical stage cost decreased to 60.5% (95% CI, 49.5%-63.1%) when failure cost was considered and to 53.0% (95% CI, 48.4%-56.9%) when the cost of capital was added. Across therapeutic areas, the expected costs for the clinical stage ranged from 53.1% (95% CI, 34.9%-63.0%) (gastrointestinal) to 66.0% (95% CI, 58.3%-67.9%) (pain and anesthesia). Similarly, the expected capitalized costs for the clinical stage ranged from 49.9% (95% CI, 38.3%-55.7%) for gastrointestinal to 55.9% (95% CI, 50.9%-59.6%) for oncology. Phase 3 cost accounted for the largest share of clinical cost primarily due to enrolling large numbers of patients (approximately 630 vs 51 for phase 1) and taking longer than phase 1 (38.0 months vs 27.8 months), on average. FDA application and review stage and postapproval costs comprised 1.5% (95% CI, 1.3%-2.0%) and 23.7% (95% CI, 17.7%-47.7%) of total mean costs, respectively.

### Trends in Pharmaceutical Industry R&D Intensity and Sales

**Figure 2.**
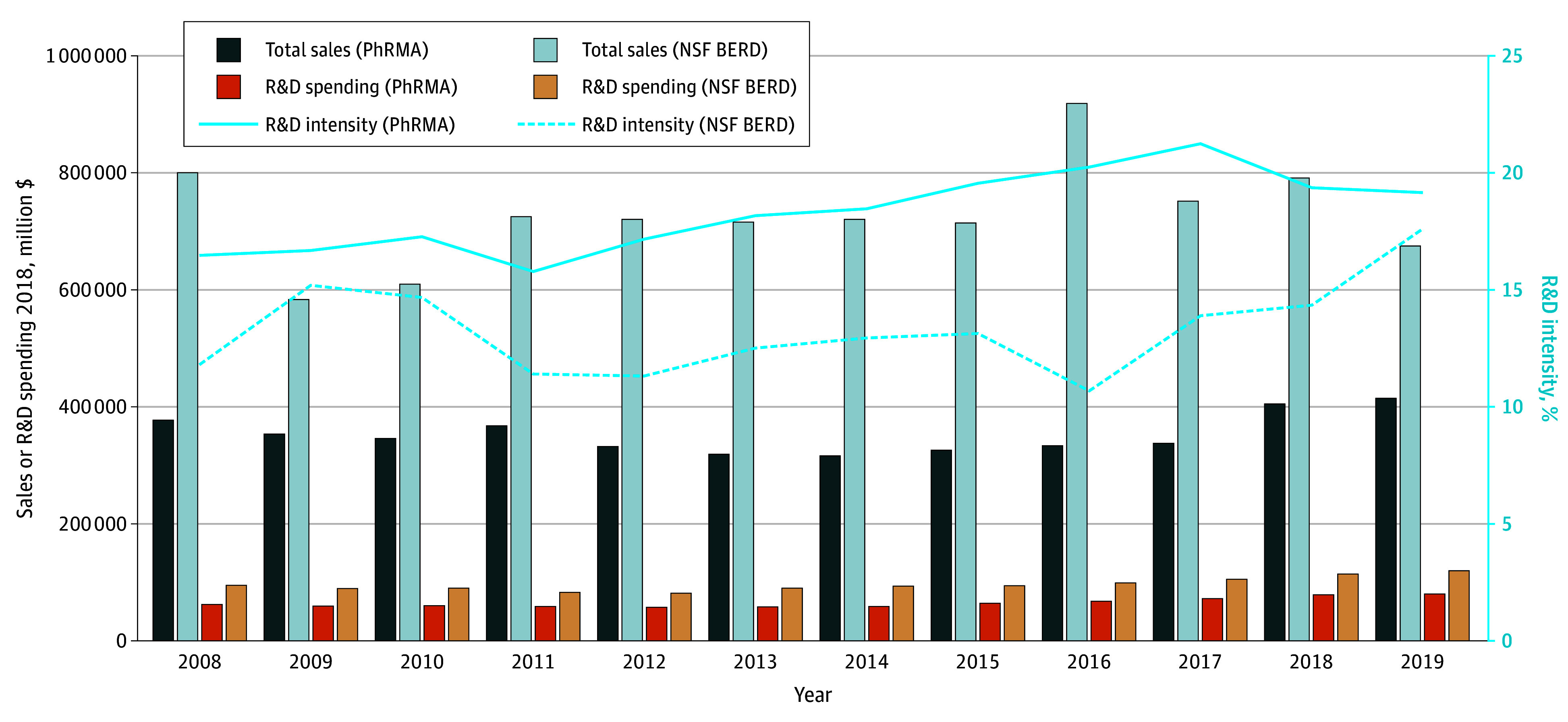
Sales, Research and Development (R&D) Spending, and R&D Intensity, 2008-2019 Data are from the National Science Foundation (NSF) Business Enterprise Research and Development Survey (BERD) and the Pharmaceutical Research and Manufacturers of America (PhRMA). Data are adjusted to real 2018 US dollars using the Medical Care Consumer Price Index.

Figure 2 shows R&D spending, sales, and R&D intensity from 2008 to 2019. Observed trends varied depending on the data source. According to BERD data, total sales for the industry as a whole have decreased by 15.6%, whereas spending on R&D has increased by 25.8%. R&D intensity has also accelerated during this period from 11.9% to 17.7%, with a mean of 13.4% for the full study period.

On the basis of PhRMA Annual Survey data,^26^ large pharmaceutical companies experienced 10.0% growth in sales (from $380.0 to $418.0 billion) and increased their R&D spending by 27.9%. R&D intensity, however, remained relatively stable, ranging from 16.6% in 2008 to 19.3% in 2019, with a mean of 18.4%. Over the 2015 to 2019 period, the R&D intensity of large pharmaceutical companies declined by 2.2% while their sales increased by 27.3%. In contrast, the industry as a whole experienced a decline of 5.5% in sales and an increase of 34.0% in R&D intensity.

## Discussion

The findings from this economic evaluation study have 2 important implications regarding strategies to control drug costs. First, although our estimates of the relative contribution of clinical phase costs to overall R&D expenditures were in line with other published findings, our estimates of cash outlays were substantially lower than those in studies that used self-reported cost data from pharmaceutical companies, which may be attributable to differences in success probability and phase duration estimates. Specifically, among those studies that reported mean cash outlays, the median cost of the clinical stage was $201.0 million (range, $47.0-$339.3 million).^28^ In this study, the mean cost of the clinical phase per drug candidate was estimated at $117.4 million, lower than the majority of the reported estimates for this stage and almost 3 times lower than the most widely cited estimate of $339.3 million by DiMasi et al,^17^ which included cost data for 1995 to 2013. Unlike many of the existing studies, where the methods and data sources are opaque or not available to facilitate replication, our study provides estimates from an analytical model developed from the bottom-up using per-patient cost figures that were based on actual negotiated contracts for clinical trials funded by pharmaceutical companies combined with parameter estimates derived from published literature via meta-analytic methods. For example, one of the key differences between our study and that of DiMasi et al^17^ is that estimates of cash outlays that are used in this model come from data that are used for benchmarking costs of clinical trials, whereas the estimates for cash outlays per drug candidate in DiMasi et al are based on industry-self-reported data that cannot be shared with external researchers.

We also found that R&D spending has increased over the 2008 to 2019 period, although its intensity remained relatively stable among large pharmaceutical companies (Figure 2). The last 5 years show particularly striking divergences in patterns between the industry as a whole and larger pharmaceutical companies. The intensity of R&D has remained relatively stable (even showed a small decline by 2.2%) among large companies despite a 27.3% growth in sales during the same period. Interestingly, the industry as a whole, which includes small and medium-sized companies, has experienced a decline of 5.5% in real sales but increased R&D intensity at 34.0% over the same 5-year period. This lends further evidence to the increasingly important role small and medium companies play in innovation; this finding is echoed in a report^29^ that showed that small and medium pharmaceutical companies accounted for 64.0% of all New Molecular Entity drug approvals in 2018.

Findings from other studies show that operating profits, particularly for larger companies, have increased steadily over the past 40 years, growing 3.5 times above their 1979 levels.^30^ Specifically, Jiang et al^30^ found an increase in operating profits from 15.3% of sales in 1979 to 23.4% of sales in 2018 for publicly-traded US pharmaceutical manufacturers. Other studies^24^ found that the profitability of large pharmaceutical companies was significantly greater compared with nonpharmaceutical companies (13.8% vs 7.7%). Taken together, these results indicate that there may be room for reducing drug prices without compromising pharmaceutical innovation, particularly for larger manufacturers.

We found that some drugs were more expensive to develop: drugs in the pain and anesthesia, oncology, and ophthalmology categories were the 3 most expensive, whereas anti-infective drugs were the least expensive. Although differences may be driven by success probabilities and clinical trial size, the cost does not correlate with market growth. For example, outpatient oral prescriptions for antibiotics grew 4.6% from 2020 to 2021.^31,32^ Further research is needed to understand strategies that align drug development and public health needs.

We found that costs associated with development failures were a major factor underlying total expected capitalized costs. Thus, strategies targeted at improving the probability of successfully moving from one stage of development to the next, such as adoption of adaptive design in clinical trials and use of biomarkers as surrogate end points, can result in substantial savings. Finally, greater transparency regarding costs incurred by pharmaceutical firms, their operating profits, and how they differ by company size would be beneficial in further understanding potential policy impacts on R&D and innovation.

### Limitations

Our study has several limitations. First, our clinical phase mean cost estimates may be underestimated due to reporting lags and lack of early-phase or non-US trial data. Second, despite the disproportionately high contribution of nonclinical stage costs to overall expected capitalized development costs, data on nonclinical phase costs are sparse. A 2012 study by Tufts Center for the Study of Drug Development^33^ estimated that nonclinical stage costs are substantially lower than the updated estimate in DiMasi et al,^17^ in which the figure was extrapolated using the ratio of total pre–human development costs to total clinical R&D spending estimated at 44.6% (an approach adopted in this study because data on nonclinical expenditures attributable to each drug candidate are not available). To the extent that actual nonclinical costs per approved drug are higher, our estimate of mean cost of drug development would be an underestimate (see eTable 7 in Supplement 1 for sensitivity analysis on nonclinical costs). Third, theoretically, the cost of capital could be lower for phase 4 studies. Thus, applying the same cost of capital to phase 4 might have resulted in overestimation of costs for that stage. Furthermore, our study, like that of DiMasi et al,^17^ did not include data on other components of costs, such as operating expenses, advertising costs, costs of goods sold, amortization, and tax expenses, which affect profitability. Further research is needed to understand the importance of such costs to R&D intensity and profitability and the factors underlying innovation. Differences in sample selection, data sources, therapeutic areas examined, definitions, and modeling assumptions may limit direct comparison with other studies.

## Conclusions

In this economic evaluation, we developed a transparent analytical model using public and proprietary sources and estimated a mean expected capitalized drug development cost of $879.3 million, which falls at the lower end of the range of other published estimates However, our estimate of mean cost (ie, the cash outlay that does not include the costs of failures or investment) was approximately 3 times lower than published findings that used data reported by pharmaceutical companies. Moreover, sales of large pharmaceutical companies have increased in recent years even as R&D intensity was stable or slightly decreasing. These results highlight the importance of understanding the scale and factors associated with the costs of drug development to inform the design of drug-related policies and their potential impacts on innovation and competition.

## Notes

Supplement 1. **eAppendix.** Supplemental Methods
eReferences
**eTable 1.** Average Per-patient Costs (in 2018 $), by Therapeutic Area and Phase **eTable 2.** Average Number of Patients per Trial, by Therapeutic Area **eTable 3.** Average Number of Trials Conducted in Support of an FDA NDA/BLA Application, by Therapeutic Area and Phase **eTable 4.** Transition Success Probabilities, by Therapeutic Area and Phase **eTable 5.** Published Estimates of Real Cost of Capital **eTable 6.** Average Phase Durations (in Months), by Therapeutic Area **eTable 7.** Sensitivity Analysis of Varying Assumptions on Non-clinical to Clinical R&D Ratio, R, on Overall Cost Estimates

Supplement 2.
Data Sharing Statement

## References

[1] Tarazi W, Finegold K, Sheingold S, Lew ND, Sommers BD. Prescription drug affordability among Medicare beneficiaries. US Department of Health and Human Services, Office of the Assistant Secretary for Planning and Evaluation. January 12, 2022. Accessed May 10, 2024. https://aspe.hhs.gov/sites/default/files/documents/1e2879846aa54939c56efeec9c6f96f0/prescription-drug-affordability.pdf

[2] Mulcahy A, Whaley C, Tebeka M, Schwam D, Edenfield N, Becerra-Ornelas A. International prescription drug price comparisons: current empirical estimates and comparisons with previous studies. RAND Corporation. January 28, 2021. Accessed May 10, 2024. https://www.rand.org/pubs/research_reports/RR2956.html

[3] Office of the Assistant Secretary for Planning and Evaluation. Comprehensive plan for addressing high drug prices: a report in response to the executive order on competition in the American economy. US Department of Health and Human Services, Office of the Assistant Secretary for Planning and Evaluation. September 9, 2021. Accessed May 10, 2024. https://aspe.hhs.gov/reports/comprehensive-plan-addressing-high-drug-prices

[4] Mulcahy AW, Schwam D, Edenfield N. Comparing insulin prices in the United States to other countries: results from a price index analysis. RAND Healthcare. October 6, 2020. Accessed May 10, 2024. https://www.rand.org/pubs/research_reports/RRA788-1.html 10.7249/rhq-11-03-03 PMC11147642 38855388

[5] Bosworth A, Sheingold S, Finegold K, Lew ND, Sommers BD. Price increases for prescription drugs, 2016-2022. US Department of Health and Human Services, Office of the Assistant Secretary for Planning and Evaluation. September 30, 2022. Accessed May 10, 2024. https://aspe.hhs.gov/reports/prescription-drug-price-increases

[6] Hernandez I, Good CB, Cutler DM, Gellad WF, Parekh N, Shrank WH. The contribution of new product entry versus existing product inflation in the rising costs of drugs. Health Aff (Millwood). 2019;38(1):76-83. doi:10.1377/hlthaff.2018.05147 30615532

[7] Philipson TJ, Durie T. The evidence base on the impact of price controls on medical innovation. University of Chicago working paper No. 2021-108. September 14, 2021. Accessed May 10, 2024. https://bfi.uchicago.edu/working-paper/the-evidence-base-on-the-impact-of-price-controls-on-medical-innovation/

[8] Committee for a Responsible Federal Budget. CBO estimates drug savings for reconciliation. July 8, 2022. Accessed October 31, 2022. https://www.crfb.org/blogs/cbo-estimates-drug-savings-reconciliation

[9] Wouters OJ, Berenbrok LA, He M, Li Y, Hernandez I. Association of research and development investments with treatment costs for new drugs approved from 2009 to 2018. JAMA Netw Open. 2022;5(9):e2218623. doi:10.1001/jamanetworkopen.2022.18623 36156148 PMC9513642

[10] DiMasi JA, Hansen RW, Grabowski HG. The price of innovation: new estimates of drug development costs. J Health Econ. 2003;22(2):151-185. doi:10.1016/S0167-6296(02)00126-1 12606142

[11] Jayasundara K, Hollis A, Krahn M, Mamdani M, Hoch JS, Grootendorst P. Estimating the clinical cost of drug development for orphan versus non-orphan drugs. Orphanet J Rare Dis. 2019;14(1):12. doi:10.1186/s13023-018-0990-4 30630499 PMC6327525

[12] Mestre-Ferrandiz J, Sussex J, Towse A. The R&D cost of a new medicine. Office of Health Economics. January 12, 2012. Accessed May 10, 2024. https://www.ohe.org/publications/rd-cost-new-medicine/

[13] Adams CP, Brantner VV. Estimating the cost of new drug development: is it really 802 million dollars? Health Aff (Millwood). 2006;25(2):420-428. doi:10.1377/hlthaff.25.2.420 16522582

[14] Adams CP, Brantner VV. Spending on new drug development. Health Econ. 2010;19(2):130-141. doi:10.1002/hec.1454 19247981

[15] DiMasi J, Grabowski H. The cost of biopharmaceutical R&D: is biotech different? Manage Decis Econ. 2007;28(4-5):469-479. doi:10.1002/mde.1360

[16] DiMasi J, Grabowski H, Vernon J. R&D costs and returns by therapeutic category. Drug Inf J. 2004;38(3):211-223. doi:10.1177/009286150403800301

[17] DiMasi JA, Grabowski HG, Hansen RW. Innovation in the pharmaceutical industry: new estimates of R&D costs. J Health Econ. 2016;47:20-33. doi:10.1016/j.jhealeco.2016.01.012 26928437

[18] Paul SM, Mytelka DS, Dunwiddie CT, . How to improve R&D productivity: the pharmaceutical industry’s grand challenge. Nat Rev Drug Discov. 2010;9(3):203-214. doi:10.1038/nrd3078 20168317

[19] Prasad V, Mailankody S. Research and development spending to bring a single cancer drug to market and revenues after approval. JAMA Intern Med. 2017;177(11):1569-1575. doi:10.1001/jamainternmed.2017.3601 28892524 PMC5710275

[20] Wouters OJ, McKee M, Luyten J. Estimated research and development investment needed to bring a new medicine to market, 2009-2018. JAMA. 2020;323(9):844-853. doi:10.1001/jama.2020.1166 32125404 PMC7054832

[21] US Government Accountability Office. Drug industry: profits, research and development spending, and merger and acquisition deals. November 17, 2017. Accessed May 10, 2024. https://www.gao.gov/products/gao-18-40

[22] Chen L. The most profitable industries in 2016. *Forbes*. December 21, 2015. Accessed December 13, 2022. https://www.forbes.com/sites/liyanchen/2015/12/21/the-most-profitable-industries-in-2016/

[23] Sood N, Shih T, Van Nuys K, Goldman D. Flow of money through the pharmaceutical distribution system. Leonard D. Schaeffer Center for Health Policy & Economics, University of Southern California. June 6, 2017. Accessed May 10, 2024. https://healthpolicy.usc.edu/research/flow-of-money-through-the-pharmaceutical-distribution-system/

[24] Ledley FD, McCoy SS, Vaughan G, Cleary EG. Profitability of large pharmaceutical companies compared with other large public companies. JAMA. 2020;323(9):834-843. doi:10.1001/jama.2020.0442 32125401 PMC7054843

[25] Rajkumar SV. The high cost of prescription drugs: causes and solutions. Blood Cancer J. 2020;10(6):71. doi:10.1038/s41408-020-0338-x 32576816 PMC7311400

[26] Pharmaceutical Research and Manufacturers of America. 2021 PhRMA annual membership survey. July 22, 2021. Accessed May 10, 2024. https://phrma.org/en/resource-center/Topics/Research-and-Development/2021-PhRMA-Annual-Membership-Survey

[27] Pharmaceutical Research and Manufacturers of America. About PhRMA. 2022. Accessed March 22, 2023. https://phrma.org/-/media/Project/PhRMA/PhRMA-Org/PhRMA-Refresh/Industry-Profile-2022/About-PhRMA-2.pdf

[28] Schlander M, Hernandez-Villafuerte K, Cheng CY, Mestre-Ferrandiz J, Baumann M. How much does it cost to research and develop a new drug? a systematic review and assessment. Pharmacoeconomics. 2021;39(11):1243-1269. doi:10.1007/s40273-021-01065-y 34368939 PMC8516790

[29] Congressional Budget Office. Research and development in the pharmaceutical industry. April 2021. Accessed May 21, 2024. https://www.cbo.gov/system/files/2021-04/57025-Rx-RnD.pdf

[30] Jiang J, Kong J, Grogan J. How did the public U.S. drugmakers’ sales, expenses and profits change over time? Leonard D. Schaeffer Center for Health Policy & Economics, University of Southern California. November 5, 2021. Accessed May 10, 2024. https://healthpolicy.usc.edu/evidence-base/how-did-the-public-u-s-drugmakers-sales-expenses-and-profits-change-over-time/

[31] Centers for Disease Control and Prevention. Outpatient antibiotic prescriptions, United States, 2020. Published 2020. Accessed May 20, 2024. https://archive.cdc.gov/www_cdc_gov/antibiotic-use/data/report-2020.html

[32] Centers for Disease Control and Prevention. Outpatient antibiotic prescriptions, United States, 2021. Published 2021. Accessed May 20, 2024. https://archive.cdc.gov/www_cdc_gov/antibiotic-use/data/report-2021.html

[33] Stergiopoulos S, Kim J, Getz K. Characterizing the cost of non-clinical development activity: understanding a critical R&D segment. Contract Pharma. June 5, 2013. Accessed May 10, 2024. https://www.contractpharma.com/issues/2013-06/view_features/characterizing-the-cost-of-non-clinical-development-activity/

