# Supplementary material for: Costs of Drug Development and Research and Development Intensity in the US, 2000-2018
Source: JAMA Netw Open. 2024 Jun 28;7(6):e2415445. doi: 10.1001/jamanetworkopen.2024.15445 (PMC11214120; doi:10.1001/jamanetworkopen.2024.15445)
Supplement: Supplement 1. — eAppendix. Supplemental Methods eReferences eTable 1. Average Per-patient Costs (in 2018 $), by Therapeutic Area and Phase eTable 2. Average Number of Patients per Trial, by Therapeutic Area eTable 3. Average Number of Trials Conducted in Support of an FDA NDA/BLA Application, by Therapeutic Area and Phase eTable 4. Transition Success Probabilities, by Therapeutic Area and Phase eTable 5. Published Estimates of Real Cost of Capital eTable 6. Average Phase Durations (in Months), by Therapeutic Area eTable 7. Sensitivity Analysis of Varying Assumptions on Non-clinical to Clinical R&D Ratio, R, on Overall Cost Estimates [file jamanetwopen-e2415445-s001.pdf]

Supplemental Online Content

Sertkaya A, Beleche T, Jessup A, Sommers BD. Costs of drug development and research and development intensity in the US, 2000-2018. *JAMA Netw Open*. 2024;7(6):e2415445. doi:10.1001/jamanetworkopen.2024.15445

eAppendix. Supplemental Methods

eReferences

eTable 1. Average Per-patient Costs (in 2018 $), by Therapeutic Area and Phase

eTable 2. Average Number of Patients per Trial, by Therapeutic Area

eTable 3. Average Number of Trials Conducted in Support of an FDA NDA/BLA Application, by Therapeutic Area and Phase

eTable 4. Transition Success Probabilities, by Therapeutic Area and Phase

eTable 5. Published Estimates of Real Cost of Capital

eTable 6. Average Phase Durations (in Months), by Therapeutic Area

eTable 7. Sensitivity Analysis of Varying Assumptions on Non-clinical to Clinical R&D Ratio, R, on Overall Cost Estimates

This supplemental material has been provided by the authors to give readers additional information about their work.

eAppendix. Supplemental Methods

The following sections discuss how expected and expected capitalized cost were calculated and the basis for the model parameter estimates presented in Table 1.

Methods

To calculate expected and expected capitalized cost, we used the method by DiMasi et al. that accounts for cost of failures and cost of capital. Figure 1 in the paper depicts the phases from early research and development to animal testing, to testing in humans, to regulatory submission for marketing approval and to post-approval studies. If the cash outlay (aka out of pocket cost) associated with a given phase i is C_i, then the expected cost, $E(C_i)$, that incorporates failures can be computed by dividing this cost by the transition success probability from phase i to launch, P_i, i.e.,

$$E(C_i) = \frac{C_i}{P_i} \quad (1)$$

where *i* = non-clinical, Phase 1, Phase 2, Phase 3, FDA review, and Phase 4.

Assuming that phase costs are distributed uniformly over the length of the phase, t_i, the capitalized cost, CC_i, that accounts for the opportunity cost of the investment in the drug is given by:

$$CC_i = \int_{t_i^e}^{t_i^b} \left(\frac{C_i}{t_i}\right) e^{rt} dt \quad (2)$$

© 2024 Sertkaya A et al. *JAMA Network Open.*

where r is the cost of capital that captures the time value effect; t_i^b is the time from the beginning, b, of the given phase to product launch, and t_i^e is the time from the end, e, of the given phase to product launch. Equation 2 then becomes:

$$CC_i = \frac{(C_i/t_i)}{r}\left(e^{rt_i^b} - e^{rt_i^e}\right) \tag{3}$$

Given equations 1 and 3, we can then compute the expected capitalized cost of phase i that accounts for the cost of failures as well as the cost of capital as:

$$E(CC_i) = \frac{CC_i}{P_i} \tag{4}$$

Then the total expected capitalized cost of development for a drug, $E(CC)$, is the sum of the expected capitalized cost of each phase i,

$$E(CC) = \sum_{i=1}^{n} E(CC_i) \tag{5}$$

Parameters

Here we present the basis for our model parameter estimates summarized in Table 1. Because our model encompasses 13 different therapeutic areas, for brevity, we generally present the overall average across all therapeutic areas, but the same approach is applied when estimating the model for each therapeutic area.

© 2024 Sertkaya A et al. *JAMA Network Open.*

Phase Durations

The phase duration parameter refers to the time it takes to complete a given stage of development. For the non-clinical stage, our estimate represents the time it takes from synthesis of the compound to the start of human trials, which includes early exploratory research for target discovery, hit generation and target identification; lead optimization; non-clinical work involving animal testing to develop dosing and toxicity models; and obtaining an Investigational New Drug (IND) approval from FDA to begin testing in human subjects. We used published studies and information compiled from FDA's DASH database to estimate average phase durations across all development stages by therapeutic area (eTable 1). Phase 3 is the longest (38.0 months) drug development stage across all therapeutic areas followed by post-approval Phase 4 (36.6 months), Phase 2 (34.0 months), non-clinical stage (31.2 months), and Phase 1 (27.8 months). The average time for the FDA review phase is 16.2 months. This includes the time the sponsor spends on responding to any questions and/or information requests from the FDA as well as preparing major/minor amendments, if needed. Thus, the estimate does not solely reflect the time FDA spends on reviewing the application. While there is variation in phase durations across the different therapeutic areas, this ranking is generally stable with Phase 3 comprising the longest stage and FDA review the shortest one.

© 2024 Sertkaya A et al. *JAMA Network Open.*

Time from Phase Start to Next Phase Start

The start-to-start parameter presented in Table 1 refers to the elapsed time between the start of one development phase (e.g., Phase 2) and the start of the next development phase (e.g., Phase 3) supporting an application. For the non-clinical phase to Phase 1 estimate, we assumed that Phase 1 will begin immediately upon successful completion of the non-clinical development phase and receipt of IND approval from FDA. Similarly, for the FDA review to approval estimate, we used the estimates reported in by therapeutic area (ranging from 9.6 months for oncology to 31.7 months for pain and anesthesia drugs). For the clinical phases, work on the clinical phases may overlap. In other words, the sponsor may begin Phase 2 clinical trials before completing the Phase 1 clinical trials. DiMasi et al. [3] estimated the average phase duration, t_i and average time to next phase, $t_{i\text{-}j}$, where i = 1, 2, 3, and j = 2, 3, FDA BLA/NDA review, for each of the three clinical phases as:

- t_1 = 33.1 months; $t_{1\text{-}2}$ = 19.8 months
- t_2 = 37.9 months; $t_{2\text{-}3}$ = 30.3 months
- t_3 = 45.1 months; $t_{3\text{-FDA BLA/NDA review}}$ = 30.7 months

To estimate the average phase-start to next phase-start durations, we used the DiMasi et al. [3] estimates along with our phase duration estimates. For example, the average Phase 1 length for the Anti-Infective therapeutic area is 21.5 months. Then, we estimated the average time to Phase 2 as the product of estimated average Phase 1 length (21.5 months) and the ratio of

© 2024 Sertkaya A et al. *JAMA Network Open.*

average time to Phase 2 to average Phase 1 length (19.8 ÷ 33.1 months) as reported in DiMasi et al. [3] at 12.9 months (= 21.5 × [19.8 ÷ 33.1] months).

There is overlap between successive stages of clinical development. For example, sponsors begin Phase 2 studies on a larger cohort of patients with more diverse conditions when initial safety and dosing results from Phase 1 studies are available even if those studies may not be fully complete. Thus, even though a Phase 1 study is estimated to last around 27.8 months on average across all therapeutic areas, a sponsor may begin a Phase 2 study on average 16.6 months after initiating the associated Phase 1 study.

Average Number of Patients Enrolled per Trial

Number of patients enrolled in a study is the largest single factor driving study costs [4]. We used three databases (Medidata, clinicaltrials.gov, and FDA DASH), of which FDA DASH and Medidata are non-public, to estimate the average number of patients enrolled per trial by therapeutic area and phase (eTable 2). The databases used cover different periods and vary in sample size, i.e., number of studies included. Ideally, the average number of patients enrolled estimate should be based on recent trials (preferably in the last 5 years) conducted in support of an NDA or BLA submission to FDA and rely on a large number of trials for each therapeutic area. None of the three databases satisfy these criteria fully. For example, Medidata database includes large number of studies, but it covers studies from 2004 through 2012 and includes trials that are not conducted in support of an NDA or BLA application to FDA. Similar to

Medidata, clinicaltrials.gov database has a large number of studies from 2014 through June 2020 but also includes those that are not conducted in support of an NDA or BLA. On the other hand, FDA DASH database includes information from more recent trials (2007 through 2017) that are conducted in support of an FDA application but has fewer studies[1] and does not include data on Phase 1 or Phase 4 trials or those trials that failed. Thus, we used all three databases to calculate the weighted average number of patients enrolled by therapeutic area and phase where the weights are the number of studies in each database.

Given the proprietary nature of information used, eTable 2 only depicts the weighted mean number of patients per trial by therapeutic area estimated, where the weights are the number of studies in each data source relative to the total number of studies across all sources.

The weighted average number of patients per trial across different therapeutic areas are highly variable. For Phase 1, the weighted average ranges from 31 patients for hematology to 121 for ophthalmology trials; 133 for dermatology to 323 immunomodulation trials for Phase 2; 233 for hematology to 1,209 for pain and anesthesia trials for Phase 3; and 261 for oncology to 1,430 for anti-infective trials for Phase 4. Across all therapeutic areas, the weighted average number of patients enrolled per trial is 51 for Phase 1, 235 for Phase 2, 630 for Phase 3, and 708 for Phase 4. Further, within several therapeutic area and phase combinations, the variation across

[1] DASH specifically captures "level of evidence" studies: pivotal and supportive studies used to support the regulatory approval of the drug. This is often a subset of the total number of trials conducted and/or submitted in the marketing application. One can then argue that since FDA DASH captures "real" applications and is a better reflection of the types of studies included in applications, then having fewer studies is not necessarily a weakness/limitation.

© 2024 Sertkaya A et al. *JAMA Network Open.*

the average number of patients reported in the different databases is also significant. For example, the average number of patients in Phase 3 cardiovascular trials in FDA DASH is over nine times larger than that estimated from clinicaltrials.gov and over five times larger than that estimated from Medidata. However, there are a few therapeutic area and phase combinations for which this variation is minimal, such as Phase 2 and Phase 3 dermatology trials.

Average Number of Trials Conducted in Support of an FDA NDA/BLA Application

Sponsors indicate whether a trial is associated with an IND when they register it in clinicaltrials.gov. However, this information is only available to the National Institutes of Health (NIH) and the FDA, not to the general public. Thus, we requested a custom data pull from FDA CDER to estimate the average number of trials per IND application. [5] FDA's internal tracking system allows drug application reviewers to select from over 800 IND Division Class Codes (Tier 3), which are mapped onto 43 broader (Tier 1) division class categories. We mapped our therapeutic areas to these 43 FDA categories and FDA CDER compiled the number of INDs and IND-linked clinical trials by these therapeutic areas and phase. Next, FDA CDER calculated the average number of trials by therapeutic area and phase by dividing the clinical trial counts for a given phase and therapeutic area by the unique IND counts for the same phase and therapeutic area. FDA CDER's estimates and model parameters are provided in eTable 3.

The average number of trials conducted in support of an FDA application for a new drug is 1.71 for Phase 1, 1.52 for Phase 2, 2.66 for Phase 3, and 1.64 for Phase 4 across all therapeutic areas.

For most therapeutic areas, sponsors conduct more than the two required Phase 3 trials with some running over four (endocrine) Phase 3 trials.

Average Cost Per Patient

eTable 4 presents the estimate of the average cost per patient used in the model. The total cost of a clinical trial for a given phase and therapeutic area, C_{total}, includes study-level costs (such as institutional review board approvals and source data verification costs), C_{study}, patient-level costs (such as recruitment and clinical procedure costs), $C_{patient}$, and site-level costs (such as monitoring and project management), C_{site} [6], i.e.:

$$C_{total} = C_{study} + C_{patient} + C_{site}$$

Then, the average cost per patient, CPP, can be calculated by dividing the total cost of a clinical trial C_{total}, by the number of patients, $n_{patient}$, enrolled in that trial, i.e.:

$$CPP = C_{total} \div n_{patient}$$

We used three different data sources to estimate the average cost per patient. Two of the data sources (Cutting Edge and Medidata) included data on total clinical trial costs and the number of patients enrolled which allowed us to directly estimate the average cost per patient using the above equation. The third source, IQVIA, only contained information on patient-level costs,

© 2024 Sertkaya A et al. *JAMA Network Open.*

which comprise between 10 to 70 percent of total trial costs depending on therapeutic area and phase according to information available from the Medidata database. For comparability, we adjusted the reported IQVIA patient-level costs by these percentages. For example, if IQVIA reported a patient level cost of $10,000 for a Phase 1 study and patient-level costs were estimated to be around 20 percent of total costs in Medidata for that therapeutic area, we estimated the IQVIA average cost per patient at $50,000 (= $10,000 ÷ 0.20). The approach assumes that the shares of study, patient, and site costs for IQVIA are equivalent to those in Medidata. Due to the proprietary nature of these databases, we only present the weighted average cost per patient estimates by therapeutic area and phase in eTable 4, where the weights are the number of studies in each database. There are no separate per-patient cost estimates available for Phase 1-2 or Phase 2-3 studies. Thus, we omitted these studies, which were only a few instances, when encountered. As expected, the average cost per patient varies significantly by therapeutic area; $19,399 (anti-infective) to $349,363 (hematology) for Phase 1, $41,323 (cardiovascular) to $100,554 (hematology) for Phase 2, $30,001 (anti-infective) to $118,473 (hematology) for Phase 3, and $13,814 (anti-infective) to $56,824 (endocrine) for Phase 4. Across all therapeutic areas, the average cost per patient is $81,338 for Phase 1, $58,618 for Phase 2, $53,180 for Phase 3, and $35,190 for Phase 4 trials.

Phase Transition Success Probabilities

The phase transition success probability parameter represents the probability of a sponsor successfully moving from one stage of drug development to the next. If, for example, out of 100

© 2024 Sertkaya A et al. *JAMA Network Open.*

new drug candidates that make it to Phase 1, 30 successfully proceed to Phase 2, then the phase transition probability from Phase 1 to Phase 2 is 30 percent. We used published studies to estimate the average phase transition success probabilities (eTable 5). Across all therapeutic areas, successfully transitioning from Phase 2 to Phase 3 generally has the lowest likelihood at 35.9 percent (ranging from 27.4 percent for respiratory system to 56.6 percent for hematology). Getting approval from the FDA for a new drug that has cleared Phase 3 has on average 88.3 percent likelihood across all therapeutic areas. Further, only 8.5 percent (= 0.68 × 0.602 × 0.359 × 0.655 × 0.883) of new drug candidates successfully move from non-clinical development to market. However, as the drug candidate successfully clears each successive development stage, the odds of making it to market improve. As expected, there is variation in this likelihood across therapeutic areas with hematology drugs having the highest likelihood at 17.8 percent and oncology drugs having the lowest likelihood at 4.1 percent (not shown).

Real Cost of Capital

The real cost of capital represents the rate of return (net of inflation) that the sponsor would otherwise be able to earn at the same risk level as the investment in the new drug that has been selected. Some critics have argued that “innovative companies must do R&D, and this is a regular cost of doing business; so estimated profits foregone should not be added to costs. If revenues are coming in from other products, then the [R&D] costs are recovered as one goes along” [7]. Others have questioned whether the appropriate cost of capital should be as high as

© 2024 Sertkaya A et al. *JAMA Network Open.*

11%, the value used in several studies from the Tufts Center for the Study of Drug Development (Tufts CSDD).

As described by Chit, et al. [8], there is an opportunity cost associated with the use of capital, which is a scarce resource, and this cost needs to be accounted for in estimating drug development costs. The value of opportunity cost of capital can vary significantly by sponsor-specific factors, such as product portfolio, venture capital funding, and size of company, as well as other exogenous factors, such as economic and regulatory climate for drug development projects. There are accepted methods in finance for estimating this cost of capital for different economic sectors and firms, including the capital asset pricing model (CAPM), and the Fama and French (F-F) 3-factor model. The CAPM model is the most widely used approach [8].

There are several CAPM studies that evaluated the real cost of capital for the pharmaceutical market as a whole as well as some broad sub-sectors, such as small and large molecules. eTable 6 presents the different the real cost of capital estimates available from the published literature. For the model, we used 11.0% as the real cost of capital for drug development projects, which is the average of figures reported for the pharmaceutical industry as a whole.

Non-clinical Stage Costs

There are no published data on non-clinical costs per drug candidate. Pharmaceutical companies have long claimed that it is difficult to attribute non-clinical R&D expenses to drug

© 2024 Sertkaya A et al. *JAMA Network Open.*

candidate compounds. In their 2016 study, DiMasi et al. estimated the ratio, R, of preclinical to clinical expenditures based on aggregated data on preclinical spending and assumptions around the duration of preclinical testing. Based on the reported amounts in Figure 2 of that study, they estimated the preclinical and clinical costs at $430 million and $965 million in 2013 dollars per approved drug, which translates to a ratio of 44.6% [3]. These estimates were based on data voluntarily submitted by anonymous biopharmaceutical companies as well as proprietary databases. The specifics of how they calculated this ratio is neither fully detailed in their study nor is available in other studies that are in the public domain. Thus, similar to other studies on this topic, we relied on the same reported ratio, 44.6%, to estimate non-clinical out of pocket costs per approved drug, which were then translated to a cost per drug candidate basis using the estimated aggregate mean success to approval rates by phase. More specifically, given that the estimated Phase 1, 2, and 3 costs are C_1, C_2, and C_3) and the estimated probability of approval from a given phase, i, is P_i, then the expected non-clinical stage cost, $E(C_{non-clinical})$, per approved drug was calculated from equation (1) as:

$$E(C_{non-clinical}) = 0.446 \times [E(C_1) + E(C_2) + E(C_3)] = 0.446 \times \left[\frac{C_1}{P_1} + \frac{C_2}{P_2} + \frac{C_3}{P_3}\right] \quad (6)$$

Then, using equations (1) and (6), the non-clinical cost per drug candidate was calculated as:

$$C_{non-clinical} = E(C_{non-clinical}) \times P_{non-clinical} \quad (7)$$

© 2024 Sertkaya A et al. *JAMA Network Open.*

Given the sizable impact of non-clinical costs on overall cost of drug development, we conducted a sensitivity analysis by varying this value +/-10% (eTable 7). As can be observed from the table, the change in this ratio results in a proportionate change in expected capitalized cost estimate but a less than proportionate change in mean cost estimate.

© 2024 Sertkaya A et al. *JAMA Network Open.*

eReferences

[1] J. A. DiMasi, H. G. Grabowski and R. W. Hansen, "Innovation in the pharmaceutical industry: new estimates of R&D costs," *Journal of health economics,* vol. 47, pp. 20-33, 2016.

[2] T. J. Moore, J. Heyward, G. Anderson and G. C. Alexander, "Variation in the estimated costs of pivotal clinical benefit trials supporting the US approval of new therapeutic agents, 2015–2017: a cross-sectional study," *BMJ Open,* vol. 10, p. e038863, 2020.

[3] U.S. Food and Drug Administration, *Custom data pull from FDA CDER's internal tracking database,* Silver Spring, MD: U.S. Food and Drug Administration, 2019d.

[4] A. Sertkaya, H.-H. Wong, A. Jessup and T. Beleche, "Key cost drivers of pharmaceutical clinical trials in the United States," *Clinical Trials,* vol. 13, no. 2, pp. 117-126, 2016.

[5] D. Light and R. Warburton, "Demythologizing the High Costs of Pharmaceutical Research," *BioSocieties,* vol. 6, pp. 34-50. doi: 10.1057/biosoc.2010.40, 2011.

[6] A. Chit, A. Chit, M. Papadimitropoulos, M. Krahn, J. Parker and P. Grootendorst, "The Opportunity Cost of Capital: Development of New Pharmaceuticals," *Inquiry: A Journal of Medical Care Organization, Provision, and Financing,* vol. 52, p. 0046958015584641. doi: 10.1177/0046958015584641, 2015.

[7] C. Wong, K. Siah and A. Lo, "Estimation of Clinical Trial Success Rates and Related Parameters," *Biostatistics,* vol. 20, no. 2, pp. 273-286, 2019.

© 2024 Sertkaya A et al. *JAMA Network Open.*

[8] BiomedTracker, "Clinical Development Success Rates 2006-2015," Biotechnology Innovation Organization, Washington, DC, 2016.

[9] U.S. Food and Drug Administration, "Custom query from FDA's DASH database," Silver Spring, MD, 2018l.

[10] R. M. Abrantes-Metz, C. P. Adams and A. Metz, "Pharmaceutical Development Phases: A Duration Analysis - Working Paper 274," Federal Trade Commission, Bureau of Economics, Washington, DC, 2004.

[11] L. Martin, M. Hutchens and C. Hawkins, "2017," *Nature Reviews: Drug Discovery,* vol. 16, no. March, p. 157, 2017.

[12] J. DiMasi, L. Feldman, A. Seckler and A. Wilson, " Trends in Risks Associated With New Drug Development: Success Rates for Investigational Drugs," *Clnical Pharmacology & Therapeutics,* vol. 87, no. 3, pp. 272-277, 2010.

[13] PAREXEL International Corp., PAREXEL Biopharmaceutical R&D Statistical Sourcebook 2017/2018, Newton, MA: PAREXEL International Corp., 2017.

[14] J. DiMasi, R. Hansen and H. Grabowski, "The Price of Innovation: New Estimates of Drug Development Costs," *Journal of Health Economics,* vol. 22, no. 2, pp. 151-185, 2003.

[15] Damodaran Online, "Data: Archives," 2018. [Online]. Available: http://people.stern.nyu.edu/adamodar/New_Home_Page/dataarchived.html. [Accessed 12 November 2019].

© 2024 Sertkaya A et al. *JAMA Network Open.*

[16] Damodaran Online, "Cost of Capital by Sector (US)," 2019. [Online]. Available: http://people.stern.nyu.edu/adamodar/New_Home_Page/datafile/wacc.htm. [Accessed 12 November 2019].

[17] S. Paul, D. Mytelka, C. Dunwiddie, C. Persinger, B. Munos, S. Lindborg and A. Schacht, "How to Improve R&D Productivity: The Pharmaceutical Industry's Grand Challenge," *Nature Reviews: Drug Discovery,* vol. 9, no. 3, pp. 203-214, 2010.

[18] S. Harrington, "Cost of Capital for Pharmaceutical, Biotechnology, and Medical Device Firms," in *The Oxford Handbook of the Economics of the Biopharmaceutical Industry*, P. Danzon and S. Nicholson, Eds., New York, Oxford University Press, 2012, pp. 75-99.

[19] L. E. Lansdowne, "Exploring the Drug Development Process," 13 March 2020. [Online]. Available: https://www.technologynetworks.com/drug-discovery/articles/exploring-the-drug-development-process-331894. [Accessed 20 July 2020].

[20] U.S. Food and Drug Administration, "The Drug Development Process," 4 January 2018b. [Online]. Available: https://www.fda.gov/patients/learn-about-drug-and-device-approvals/drug-development-process. [Accessed 17 July 2020].

[21] J. DiMasi, R. G. H. Hansen and L. Lasagna, "Cost of innovation in the pharmaceutical industry," *Journal of Health Economics,* vol. 10, pp. 107-142, 1991.

© 2024 Sertkaya A et al. *JAMA Network Open.*

eTable 1. Average Per-patient Costs (in 2018 $), by Therapeutic Area and Phase

Therapeutic Area	Phase	Weighted Average
Anti-Infective	Phase 1	$19,399
	Phase 2	$59,289
	Phase 3	$30,001
	Phase 4	$13,814
Cardiovascular	Phase 1	$59,456
	Phase 2	$41,323
	Phase 3	$33,084
	Phase 4	$33,915
Central Nervous System	Phase 1	$87,390
	Phase 2	$48,767
	Phase 3	$39,612
	Phase 4	$34,956
Dermatology	Phase 1	$35,450
	Phase 2	$66,661
	Phase 3	$48,587
	Phase 4	$33,102
Endocrine	Phase 1	$85,463
	Phase 2	$51,556
	Phase 3	$48,753
	Phase 4	$56,824
Gastrointestinal	Phase 1	$61,848
	Phase 2	$63,590
	Phase 3	$47,656
	Phase 4	$52,746
Genitourinary System	Phase 1	$53,770
	Phase 2	$45,781
	Phase 3	$38,930
	Phase 4	$16,699
Hematology [a]	Phase 1	$349,363
	Phase 2	$100,554
	Phase 3	$118,473

© 2024 Sertkaya A et al. *JAMA Network Open.*

Therapeutic Area	Phase	Weighted Average
	Phase 4	$41,958
Immunomodulation	Phase 1	$63,471
	Phase 2	$47,897
	Phase 3	$54,909
	Phase 4	$30,246
Oncology	Phase 1	$103,344
	Phase 2	$78,753
	Phase 3	$93,145
	Phase 4	$23,515
Ophthalmology	Phase 1	$50,999
	Phase 2	$48,438
	Phase 3	$79,933
	Phase 4	$24,022
Pain and Anesthesia	Phase 1	$90,370
	Phase 2	$77,726
	Phase 3	$60,751
	Phase 4	$41,573
Respiratory System	Phase 1	$44,330
	Phase 2	$43,563
	Phase 3	$46,764
	Phase 4	$18,987
**All Therapeutic Areas**	**Phase 1**	**$81,338**
	**Phase 2**	**$58,618**
	**Phase 3**	**$53,180**
	**Phase 4**	**$35,190**

[a] The representativeness of this category is highly limited due to small sample sizes and the types of indications covered in the included trials.

© 2024 Sertkaya A et al. *JAMA Network Open.*

eTable 2. Average Number of Patients per Trial, by Therapeutic Area

Therapeutic Area	Phase	Weighted Average Number of Patients per Trial
Anti-Infective	Phase 1	69
	Phase 2	243
	Phase 3	575
	Phase 4	1,430
Cardiovascular	Phase 1	42
	Phase 2	189
	Phase 3	1,151
	Phase 4	508
Central Nervous System	Phase 1	44
	Phase 2	243
	Phase 3	529
	Phase 4	356
Dermatology	Phase 1	106
	Phase 2	133
	Phase 3	568
	Phase 4	850
Endocrine	Phase 1	38
	Phase 2	225
	Phase 3	414
	Phase 4	482
Gastrointestinal	Phase 1	38
	Phase 2	292
	Phase 3	496
	Phase 4	1,344
Genitourinary System	Phase 1	50
	Phase 2	323
	Phase 3	546
	Phase 4	410
Hematology	Phase 1	31
	Phase 2	134
	Phase 3	233
	Phase 4	411
Immunomodulation	Phase 1	55
	Phase 2	323
	Phase 3	309
	Phase 4	383
Oncology	Phase 1	58
	Phase 2	137
	Phase 3	293
	Phase 4	261
Ophthalmology	Phase 1	121
	Phase 2	299
	Phase 3	876
	Phase 4	413
Pain and Anesthesia	Phase 1	36

© 2024 Sertkaya A et al. *JAMA Network Open.*

Therapeutic Area	Phase	Weighted Average Number of Patients per Trial
	Phase 2	270
	Phase 3	1,209
	Phase 4	280
Respiratory System	Phase 1	49
	Phase 2	203
	Phase 3	516
	Phase 4	1,159
**All Therapeutic Areas**	**Phase 1**	**51**
	**Phase 2**	**235**
	**Phase 3**	**630**
	**Phase 4**	**708**

© 2024 Sertkaya A et al. *JAMA Network Open.*

eTable 3. Average Number of Trials Conducted in Support of an FDA NDA/BLA Application, by Therapeutic Area and Phase

Therapeutic Area	Phase 1	Phase 2	Phase 3	Phase 4
Anti-Infective	2.06	1.55	2.41	1.55
Cardiovascular	1.65	1.43	2.40	1.99
Central Nervous System	1.77	1.36	2.83	1.56
Dermatology	1.74	1.69	2.56	1.42
Endocrine	2.11	1.67	4.25	1.91
Gastrointestinal	1.81	1.41	2.46	1.61
Genitourinary System	1.58	1.34	1.47	1.00
Hematology	1.62	1.63	2.37	1.59
Immunomodulation	1.96	1.57	3.09	1.86
Oncology	1.36	1.34	1.63	1.29
Ophthalmology	1.23	1.57	2.47	1.83
Pain and Anesthesia	1.90	1.65	2.92	1.49
Respiratory System	1.46	1.55	3.75	2.22
**All Therapeutic Areas**	**1.71**	**1.52**	**2.66**	**1.64**

Source: FDA CDER, [5]

[a] Data are current as of 7/23/2019.

[b] Excludes INDs received by CDER prior to the establishment of clinicaltrials.gov.

[c] Excludes trials not conducted under an IND.

[d] Excludes trials not registered with clinicaltrials.gov.

[e] Excludes trials associated with INDs not having a Division Classification Code.

[f] Excludes trials associated with INDs having a Division Classification Code that was not mapped to any of the therapeutic areas included in this model

[g] Division Classification Codes have not undergone quality control to ensure accuracy.

[h] The figures are calculated by dividing the number of trials for a given therapeutic area and phase by a distinct count of IND(s) associated with the corresponding cohort of trials (within the same therapeutic area and phase).

© 2024 Sertkaya A et al. *JAMA Network Open.*

eTable 4. Transition Success Probabilities, by Therapeutic Area and Phase

Data Source	Time Period	Therapeutic Area	Therapeutic Area in the Original Source [a]	Other Classification	Non-clinical to Phase 1	Phase 1 to Phase 2	Phase 2 to Phase 3	Phase 3 to FDA Review	FDA Review to Approval
Wong et al [9]	2000 - 2015	Anti-Infective	Infectious Disease	NA	NA	70.1%	58.3%	NA	NA
DiMasi et al [14]	1993-2004		Systemic Anti-infective	NA	NA	58.2%	52.2%	78.6%	100.0%
BiomedTracker [10]	2006-2015		Infectious Disease	NA	NA	69.5%	42.7%	72.7%	88.7%
BiomedTracker, 2017 [c]	2010-2016		Infectious Disease	NA	NA	NA	45.0%	71.0%	NA
Wong et al [9]	2000 - 2015	Cardiovascular	Cardiovascular	NA	NA	73.3%	65.7%	NA	NA
DiMasi et al [14]	1993-2004		Cardiovascular	NA	NA	62.9%	32.4%	64.3%	66.7%
BiomedTracker [10]	2006-2015		Cardiovascular	NA	NA	58.9%	24.1%	55.5%	84.2%
BiomedTracker, 2017 [c]	2010-2016		Cardiovascular	NA	NA	NA	26.0%	53.0%	NA
Wong et al [9]	2000 - 2015	Central Nervous System	Central Nervous System	NA	NA	73.2%	51.9%	NA	NA
DiMasi et al [14]	1993-2004		Central Nervous System	NA	NA	59.6%	33.0%	46.4%	90.0%
BiomedTracker [10]	2006-2015		Neurology	NA	NA	59.1%	29.7%	57.4%	83.2%
BiomedTracker [10]	2006-2015		Psychiatry	NA	NA	53.9%	23.7%	55.7%	87.9%
BiomedTracker, 2017 [c]	2010-2016		Neurology	NA	NA	NA	33.0%	60.0%	NA
BiomedTracker, 2017 [c]	2010-2016		Psychiatry	NA	NA	NA	27.0%	60.0%	NA
Wong et al [9]	2000 - 2015	Endocrine	Metabolic Diseases	NA	NA	76.2%	59.7%	NA	NA
Wong et al [9]	2000 - 2015		Endocrinology	NA	NA	76.2%	59.7%	NA	NA
DiMasi et al [14]	1993-2004		Gastroenterology/Metabolism	NA	NA	67.5%	34.9%	50.0%	80.0%
BiomedTracker [10]	2006-2015		Metabolic Diseases	NA	NA	61.1%	45.2%	71.4%	77.8%
BiomedTracker [10]	2006-2015		Endocrinology	NA	NA	58.9%	40.1%	65.0%	86.0%
BiomedTracker, 2017 [c]	2010-2016		Endocrinology	NA	NA	NA	38.0%	69.0%	NA
DiMasi et al [14]	1993-2004	Gastrointestinal	Gastroenterology/Metabolism	NA	NA	67.5%	34.9%	50.0%	80.0%
BiomedTracker [10]	2006-2015		Gastroenterology	NA	NA	75.6%	35.7%	60.6%	92.3%
Wong et al [9]	2000 - 2015	Genitourinary System	Genitourinary	NA	NA	68.7%	57.1%	NA	NA
BiomedTracker [10]	2006-2015		Urology	NA	NA	57.1%	32.7%	71.4%	85.7%
BiomedTracker [10]	2006-2015	Hematology	Hematology	NA	NA	73.3%	56.6%	75.0%	84.0%
Wong et al [9]	2000 - 2015	Immunomodulation	Autoimmune Diseases	NA	NA	69.8%	45.7%	NA	NA
Wong et al [9]	2000 - 2015		Inflammation	NA	NA	69.8%	45.7%	NA	NA
DiMasi et al [14]	1993-2004		Antineoplastic/immunologic	NA	NA	71.8%	49.0%	55.3%	100.0%
DiMasi et al [14]	1993-2004		Musculoskeletal	NA	NA	72.4%	35.2%	80.0%	100.0%
BiomedTracker [10]	2006-2015		Autoimmune Diseases	NA	NA	65.7%	31.7%	62.2%	86.0%
BiomedTracker, 2017	2010-2016		Autoimmune Diseases	NA	NA	NA	33.0%	64.0%	NA
Wong et al [9]	2000 - 2015	Oncology	Oncology	NA	NA	57.6%	32.7%	NA	NA
DiMasi et al [14]	1993-2004		Antineoplastic/immunologic	NA	NA	71.8%	49.0%	55.3%	100.0%

© 2024 Sertkaya A et al. *JAMA Network Open.*

Data Source	Time Period	Therapeutic Area	Therapeutic Area in the Original Source [a]	Other Classification	Non-clinical to Phase 1	Phase 1 to Phase 2	Phase 2 to Phase 3	Phase 3 to FDA Review	FDA Review to Approval
BiomedTracker [10]	2006-2015		Oncology	NA	NA	62.8%	24.6%	40.1%	82.4%
BiomedTracker [10]	2006-2015		Solid Tumors	NA	NA	64.1%	23.0%	34.2%	79.6%
BiomedTracker [10]	2006-2015		Hematological Cancers	NA	NA	61.8%	28.7%	52.6%	86.4%
BiomedTracker, 2017 [c]	2010-2016		Oncology	NA	NA	NA	27.0%	45.0%	NA
Pharma Intelligence, Informa, 2016 [c]	2011-2015		Oncology	NA	NA	59.0%	21.0%	38.0%	84.0%
Pharma Intelligence, Informa, 2016 [c]	2011-2015		Solid Tumors	NA	NA	57.0%	20.0%	32.0%	83.0%
Pharma Intelligence, Informa, 2016 [c]	2011-2015		Hematological Cancers	NA	NA	64.0%	26.0%	54.0%	84.0%
Pharma Intelligence, Informa, 2016 [c]	2011-2015		Oncology	NME	NA	56.0%	18.0%	36.0%	77.0%
Pharma Intelligence, Informa, 2016 [c]	2011-2015		Oncology	Large Molecule	NA	61.0%	25.0%	40.0%	93.0%
Wong et al [9]	2000 - 2015	Ophthalmology	Ophthalmology	NA	NA	87.1%	60.7%	NA	NA
BiomedTracker [10]	2006-2015		Ophthalmology	NA	NA	84.8%	44.6%	58.3%	77.5%
DiMasi et al [14]	1993-2004	Respiratory System	Respiratory	NA	NA	72.5%	20.0%	85.7%	80.0%
BiomedTracker [10]	2006-2015		Allergy	NA	NA	67.6%	32.5%	71.4%	93.8%
BiomedTracker [10]	2006-2015		Respiratory	NA	NA	65.3%	29.1%	71.1%	94.6%
BiomedTracker, 2017 [c]	2010-2016		Respiratory	NA	NA	NA	28.0%	74.0%	NA
Wong et al [9]	2000 - 2015	All Therapeutic Areas	Overall	NA	NA	66.4%	58.3%	NA	NA
DiMasi et al [14]	1993-1998		Overall	NA	NA	67.0%	41.0%	63.0%	90.0%
DiMasi et al [14]	1999-2004		Overall	NA	NA	64.0%	39.0%	66.0%	100.0%
DiMasi et al [14]	1993-2004		Overall	Self-originated	NA	65.0%	40.0%	64.0%	93.0%
DiMasi et al [14]	1993-2004		Overall	Licensed-in	NA	82.0%	56.0%	64.0%	93.0%
DiMasi et al [14]	1993-2004		Overall	NA	NA	71.0%	45.0%	64.0%	93.0%
DiMasi et al [14]	1993-2004		Overall	Small Molecule	NA	63.0%	38.0%	61.0%	91.0%
DiMasi et al [14]	1993-2004		Overall	Large Molecule	NA	84.0%	53.0%	74.0%	96.0%
DiMasi et al [14]	1995-2007		Overall	NA	NA	59.5%	35.5%	62.0%	90.4%
BiomedTracker [10]	2006-2015		Overall	NA	NA	63.2%	30.7%	58.1%	85.3%
BiomedTracker [10]	2006-2015		Overall	NME	NA	61.3%	26.5%	48.7%	78.0%
BiomedTracker [10]	2006-2015		Overall	Large Molecule	NA	66.0%	34.4%	57.2%	88.4%
BiomedTracker [10]	2006-2015		Overall	Non-NME	NA	70.1%	48.3%	73.9%	90.4%
BiomedTracker [10]	2006-2015		Chronic High Prevalence Diseases	NA	NA	58.7%	27.7%	61.6%	87.2%
BiomedTracker [10]	2006-2015		Rare Diseases	NA	NA	76.0%	50.6%	73.6%	89.2%
KMR Bernstein Analysis, 2016 [c]	2003-2007		Overall	NA	69.0%	54.0%	34.0%	70.0%	91.0%
KMR Bernstein Analysis, 2016 [c]	2005-2009		Overall	NA	64.0%	48.0%	25.0%	67.0%	83.0%
KMR Bernstein Analysis, 2016 [c]	2007-2011		Overall	NA	64.0%	44.0%	22.0%	65.0%	83.0%
KMR Bernstein Analysis, 2016 [c]	2007-2011		Overall	Small Molecule	61.0%	42.0%	18.0%	60.0%	85.0%

© 2024 Sertkaya A et al. *JAMA Network Open.*

Data Source	Time Period	Therapeutic Area	Therapeutic Area in the Original Source [a]	Other Classification	Non-clinical to Phase 1	Phase 1 to Phase 2	Phase 2 to Phase 3	Phase 3 to FDA Review	FDA Review to Approval
KMR Bernstein Analysis, 2016 [c]	2007-2011		Overall	Large Molecule	75.0%	56.0%	44.0%	79.0%	79.0%
KMR Bernstein Analysis, 2016 [c]	2010-2014		Overall	NA	67.0%	44.0%	29.0%	69.0%	88.0%
KMR Bernstein Analysis, 2016 [c]	2010-2014		Overall	Small Molecule	62.0%	40.0%	24.0%	65.0%	90.0%
KMR Bernstein Analysis, 2016 [c]	2010-2014		Overall	Large Molecule	76.0%	53.0%	40.0%	79.0%	86.0%
KMR Bernstein Analysis, 2016 [c]	2011-2015		Overall	NA	68.0%	45.0%	33.0%	77.0%	92.0%
KMR Bernstein Analysis, 2016 [c]	2011-2015		Overall	Small Molecule	63.0%	41.0%	30.0%	72.0%	92.0%
KMR Bernstein Analysis, 2016 [c]	2011-2015		Overall	Large Molecule	79.0%	52.0%	39.0%	88.0%	93.0%
BiomedTracker, 2017 [c]	2011		Overall	NA	NA	64.0%	32.0%	60.0%	83.0%
BiomedTracker, 2017 [c]	2012		Overall	NA	NA	65.0%	33.0%	61.0%	85.0%
BiomedTracker, 2017 [c]	2013		Overall	NA	NA	64.0%	32.0%	60.0%	86.0%
BiomedTracker, 2017 [c]	2014		Overall	NA	NA	65.0%	33.0%	62.0%	87.0%
BiomedTracker, 2017 [c]	2015		Overall	NA	NA	64.0%	32.0%	61.0%	87.0%
BiomedTracker, 2017 [c]	2016		Overall	NA	NA	65.0%	33.0%	60.0%	88.0%
BiomedTracker, 2017 [c]	2016		Overall	NME	NA	62.0%	28.0%	51.0%	83.0%
**Anti-Infective Average**					**68.0% [b]**	**65.9%**	**49.6%**	**74.1%**	**94.4%**
**Cardiovascular Average**						**65.0%**	**37.1%**	**57.6%**	**75.5%**
**Central Nervous System Average**						**61.5%**	**33.1%**	**55.9%**	**87.0%**
**Dermatology Average**						**60.2% [b]**	**35.9% [b]**	**65.5% [b]**	**88.3% [b]**
**Endocrine Average**						**68.0%**	**46.3%**	**63.9%**	**81.3%**
**Gastrointestinal Average**						**71.6%**	**35.3%**	**55.3%**	**86.2%**
**Genitourinary System Average**						**62.9%**	**44.9%**	**71.4%**	**85.7%**
**Hematology Average**						**73.3%**	**56.6%**	**75.0%**	**84.0%**
**Oncology Average**						**61.5%**	**26.8%**	**42.7%**	**85.5%**
**Respiratory System Average**						**68.5%**	**27.4%**	**75.6%**	**89.5%**
**Ophthalmology Average**						**86.0%**	**52.7%**	**58.3%**	**77.5%**
**Pain and Anesthesia Average**						**60.2% [b]**	**35.9% [b]**	**65.5% [b]**	**88.3% [b]**
**Immunomodulation Average**						**69.9%**	**40.1%**	**65.4%**	**95.3%**
**All Therapeutic Areas Average**						**60.2%**	**35.9%**	**65.5%**	**88.3%**

NA = Not available/Not applicable
[a] This represents the therapeutic area or disease for which the duration estimates correspond to in the original source. We mapped these reported therapeutic areas and/or diseases to the therapeutic areas in this model.
[b] The figure is the All Therapeutic Areas average transition probability as no information was available for the therapeutic area and phase-to-phase combination.
[c] From PAREXEL's biopharmaceutical R&D statistical yearbook [15].

© 2024 Sertkaya A et al. *JAMA Network Open.*

eTable 5. Published Estimates of Real Cost of Capital

Data Source	Sub-Sector	Firm Size	Study Period	Sample Size	Opportunity Cost of Capital
DiMasi et al, [3]	All	All	2000	NA	11.8%
			2005	NA	10.8%
			2010	NA	9.4%
DiMasi et al, [16]	All	All	2000	NA	11.9%
Damodaran, [17]	Large Molecule	All	2018	459	9.2%
	Small Molecule	All	2018	185	8.1%
Damodaran, [18]	Large Molecule	All	2019	481	10.5%
	Small Molecule	All	2019	237	10.5%
Paul et al, [19]	All	All	2007	NA	11.0%
Harrington, [20]	Small Molecule	All	2001-2005	31	9.8%
		Large	2001-2005	22	9.6%
		Small	2001-2005	9	10.6%
	Large Molecule	All	2001-2005	26	14.2%
		Large	2001-2005	17	14.1%
		Small	2001-2005	9	14.5%
	Small Molecule	All	2006-2008	28	9.3%
		Large	2006-2008	21	9.5%
		Small	2006-2008	7	8.6%
	Large Molecule	All	2006-2008	29	11.8%
		Large	2006-2008	14	10.2%
		Small	2006-2008	15	13.2%
**Average**	**Large Molecule**	**Large**			**12.2%**
		**Small**			**13.9%**
		**All**			**11.4%**
	**Small Molecule**	**Large**			**9.6%**
		**Small**			**9.6%**
		**All**			**9.4%**
	**All**	**Large**			**NA**
		**Small**			**NA**
		**All [a]**			**11.0%**

NA = Not available
[a] Estimate used in this model.

eTable 6. Average Phase Durations (in Months), by Therapeutic Area

Source	Time Period	Therapeutic Area	Therapeutic Area in the Original Source [a]	Non-clinical	Phase 1	Phase 2	Phase 3	FDA BLA/NDA Review	Phase 4
Wong et al [9]	2000-2015	Anti-Infective	Infectious Disease	NA	18.4	31.2	35.0	NA	38.7
BiomedTracker [10]	2006-2015		Infectious Disease	NA	NA	NA	NA	16.8	NA
FDA DASH Query [11]	2007-2017		Anti-Infective	NA	NA	NA	NA	12.9	NA
Abrantes-Metz et al [12]	1989-2002		Anti-HIV/AIDS	NA	24.7	24.8	30.6	NA	NA
Wong et al [9]	2000-2015	Cardiovascular	Cardiovascular	NA	12.4	33.6	39.6	NA	38.5
BiomedTracker [10]	2006-2015		Cardiovascular	NA	NA	NA	NA	16.8	NA
FDA DASH Query [11]	2007-2017		Cardiovascular	NA	NA	NA	NA	21.5	NA
Abrantes-Metz et al [12]	1989-2002		Anti-Hypertension	NA	15.9	42.5	44.4	NA	NA
Wong et al [9]	2000-2015	Central Nervous System	Central Nervous System	NA	11.0	30.6	33.9	NA	35.0
BiomedTracker [10]	2006-2015		Neurology	NA	NA	NA	NA	23.9	NA
BiomedTracker [10]	2006-2015		Psychiatry	NA	NA	NA	NA	19.1	NA
FDA DASH Query [11]	2007-2017		Central Nervous System	NA	NA	NA	NA	19.8	NA
Abrantes-Metz et al [12]	1989-2002		Anti-Alzheimer's Disease	NA	23.2	46.9	41.8	NA	NA
Abrantes-Metz et al [12]	1989-2002		Anti-Parkinson's Disease	NA	24.4	42.9	60.1	NA	NA
FDA DASH Query [11]	2007-2017	Dermatology	Dermatology	NA	NA	NA	NA	12.2	NA
Wong et al [9]	2000-2015	Endocrine	Metabolic Diseases	NA	10.7	31.0	32.0	NA	34.0
Wong et al [9]	2000-2015		Endocrinology	NA	10.7	31.0	32.0	NA	34.0
BiomedTracker [10]	2006-2015		Metabolic Diseases	NA	NA	NA	NA	18.0	NA
BiomedTracker [10]	2006-2015		Endocrinology	NA	NA	NA	NA	21.5	NA
FDA DASH Query [11]	2007-2017		Endocrine	NA	NA	NA	NA	16.9	NA
Abrantes-Metz et al [12]	1989-2002		Anti-Diabetes	NA	17.4	25.8	42.7	NA	NA

© 2024 Sertkaya A et al. *JAMA Network Open.*

Source	Time Period	Therapeutic Area	Therapeutic Area in the Original Source [a]	Non-clinical	Phase 1	Phase 2	Phase 3	FDA BLA/NDA Review	Phase 4
BiomedTracker [10]	2006-2015	Gastrointestinal	Gastroenterology	NA	NA	NA	NA	21.5	NA
FDA DASH Query [11]	2007-2017		Gastrointestinal	NA	NA	NA	NA	14.2	NA
Wong et al [9]	2000-2015	Genitourinary System	Genitourinary	NA	12.4	25.8	33.0	NA	29.9
BiomedTracker [10]	2006-2015		Urology	NA	NA	NA	NA	20.4	NA
FDA DASH Query [11]	2007-2017		Genitourinary System	NA	NA	NA	NA	16.2	NA
BiomedTracker [10]	2006-2015	Hematology	Hematology	NA	NA	NA	NA	19.1	NA
FDA DASH Query [11]	2007-2017		Hematology	NA	NA	NA	NA	11.5	NA
Abrantes-Metz et al [12]	1989-2002		Anti-Thrombosis	NA	23.8	35.0	58.3	NA	NA
Wong et al [9]	2000-2015	Immunomodulation	Autoimmune Diseases	NA	11.0	32.1	32.1	NA	39.6
Wong et al [9]	2000-2015		Inflammation	NA	11.0	32.1	32.1	NA	39.6
BiomedTracker [10]	2006-2015		Autoimmune Diseases	NA	NA	NA	NA	19.1	NA
FDA DASH Query [11]	2007-2017		Immunomodulation	NA	NA	NA	NA	14.5	NA
Abrantes-Metz et al [12]	1989-2002		Anti-Arthritis	NA	17.5	35.1	44.8	NA	NA
Wong et al [9]	2000-2015	Oncology	Oncology	NA	39.9	48.9	68.2	NA	45.7
BiomedTracker [10]	2006-2015		Oncology	NA	NA	NA	NA	13.2	NA
FDA DASH Query [11]	2007-2017		Oncology	NA	NA	NA	NA	5.9	NA
Abrantes-Metz et al [12]	1989-2002		Anti-Cancer	NA	23.8	31.7	47.1	NA	NA
Wong et al [9]	2000-2015	Ophthalmology	Ophthalmology	NA	17.9	27.0	33.7	NA	30.7
BiomedTracker [10]	2006-2015		Ophthalmology	NA	NA	NA	NA	15.6	NA
FDA DASH Query [11]	2007-2017		Ophthalmology	NA	NA	NA	NA	8.3	NA
FDA DASH Query [11]	2007-2017	Pain and Anesthesia	Pain and Anesthesia	NA	NA	NA	NA	31.7	NA
BiomedTracker [10]	2006-2015	Respiratory System	Allergy	NA	NA	NA	NA	15.6	NA
BiomedTracker [10]	2006-2015		Respiratory	NA	NA	NA	NA	19.1	NA

© 2024 Sertkaya A et al. *JAMA Network Open.*

Source	Time Period	Therapeutic Area	Therapeutic Area in the Original Source [a]	Non-clinical	Phase 1	Phase 2	Phase 3	FDA BLA/NDA Review	Phase 4
FDA DASH Query [11]	2007-2017		Respiratory System	NA	NA	NA	NA	21.9	NA
Abrantes-Metz et al [12]	1989-2002		Anti-Asthma	NA	17.6	37.1	42.2	NA	NA
Wong et al [9]	2000-2015		Overall	NA	19.3	35.2	46.0	NA	NA
BiomedTracker [10]	2006-2015		Overall	NA	NA	NA	NA	19.1	NA
FDA DASH Query [11]	2007-2017		Overall	NA	NA	NA	NA	13.6	NA
Martin, Hutchens, & Hawkins [13]	2006-2008		Overall	NA	30.9	32.9	32.9	NA	NA
Martin, Hutchens, & Hawkins [13]	2007-2009		Overall	NA	32.9	32.9	34.9	NA	NA
Martin, Hutchens, & Hawkins [13]	2008-2010		Overall	NA	30.9	33.9	34.9	NA	NA
Martin, Hutchens, & Hawkins [13]	2009-2011	All Therapeutic Areas	Overall	NA	30.9	33.9	33.9	NA	NA
Martin, Hutchens, & Hawkins [13]	2010-2012		Overall	NA	27.0	34.9	36.9	NA	NA
Martin, Hutchens, & Hawkins [13]	2011-2013		Overall	NA	29.0	32.9	41.9	NA	NA
Martin, Hutchens, & Hawkins [13]	2012-2014		Overall	NA	30.9	35.9	40.9	NA	NA
Martin, Hutchens, & Hawkins [13]	2013-2015		Overall	NA	31.9	39.9	38.9	NA	NA
DiMasi, Grabowski, Hansen, [3]	NA		Overall	31.2	19.8	30.3	30.7	16.0	NA
Abrantes-Metz et al [12]	1989-2002		Overall	NA	22.0	31.6	45.9	NA	NA
**Anti-Infective Average**					**21.5**	**28.0**	**32.8**	**14.8**	**38.7**
**Cardiovascular Average**					**14.1**	**38.0**	**42.0**	**19.1**	**38.5**
**Central Nervous System Average**					**19.5**	**40.1**	**45.3**	**21.0**	**35.0**
**Dermatology Average**					**27.8 [b]**	**34.0 [b]**	**38.0 [b]**	**12.2**	**36.6 [b]**
**Endocrine Average**					**12.9**	**29.3**	**35.6**	**18.8**	**34.0**
**Gastrointestinal Average**					**27.8 [b]**	**34.0 [b]**	**38.0 [b]**	**17.9**	**36.6 [b]**
**Genitourinary System Average**					**12.4**	**25.8**	**33.0**	**18.3**	**29.9**
**Hematology Average**				**31.2 [b]**	**23.8**	**35.0**	**58.3**	**15.3**	**36.6 [b]**
**Oncology Average**					**31.9**	**40.3**	**57.7**	**9.6**	**45.7**
**Respiratory System Average**					**17.6**	**37.1**	**42.2**	**18.9**	**36.6**
**Ophthalmology Average**					**17.9**	**27.0**	**33.7**	**11.9**	**30.7**
**Pain and Anesthesia Average**					**27.8 [b]**	**34.0 [b]**	**38.0 [b]**	**31.7**	**36.6 [b]**
**Immunomodulation Average**					**13.1**	**33.1**	**36.3**	**16.8**	**39.6**
**All Therapeutic Areas Average**					**27.8**	**34.0**	**38.0**	**16.2**	**36.6] [c]**

NA = Not available

© 2024 Sertkaya A et al. *JAMA Network Open.*

Source	Time Period	Therapeutic Area	Therapeutic Area in the Original Source [a]	Non-clinical	Phase 1	Phase 2	Phase 3	FDA BLA/NDA Review	Phase 4

[a] This represents the therapeutic area or disease for which the duration estimates correspond to in the original source. We mapped these reported therapeutic areas and/or diseases to the therapeutic areas in this model.
[b] The figure is the All Therapeutic Areas average duration as no information was available for the therapeutic area and phase combination.
[c] This represents the average across all estimates in the table from Anti-infective through Immunomodulation therapeutic areas.

eTable 7. Sensitivity Analysis of Varying Assumptions on Non-clinical to Clinical R&D Ratio, R, on Overall Cost Estimates

Scenario	Cost ($) [a]	Expected Cost ($) [b]	Expected Capitalized Cost ($) [c]
Base Case: R = 44.6%	172.7<br>(132.5 - 197.9)	515.8<br>(327.0 - 773.2)	879.3<br>(416.9 – 1,307.3)
Scenario 1: R = 34.6%	170.0<br>(131.0 – 194.6)	484.6<br>(309.0 – 731.0)	800.0<br>(380.0 – 1,201.0)
Scenario 2: R = 54.6%	175.3<br>(134.2 – 201.1)	547.1<br>(342.0 – 816.0)	958.7<br>(455.0 – 1,441.0)
Scenario 3: R ~ *N*(44.6%, 10%)	172.7<br>(132.8 – 199.5)	515.8<br>(322.0 – 778.0)	879.3<br>(407.0 – 1,348.0)

[a] Represents the cash outlay not adjusted for the cost of capital or failures.
[b] Represents R&D cost after adjusting for the cost of failures computed as the total cash outlay divided by the aggregate transition success probability; includes cost of failures but not the cost of capital.
[c] Represents costs inclusive of failure and capital costs.

© 2024 Sertkaya A et al. *JAMA Network Open.*
