# Supplementary material for: Costs of Drug Development and Research and Development Intensity in the US, 2000-2018
Source: JAMA Netw Open. 2024 Jun 28;7(6):e2415445. doi: 10.1001/jamanetworkopen.2024.15445 (PMC11214120; doi:10.1001/jamanetworkopen.2024.15445)
Supplement: Supplement 2. — Data Sharing Statement [file jamanetwopen-e2415445-s002.pdf]

Data Sharing Statement

Sertkaya. Costs of Drug Development and Research and Development Intensity in the US, 2000-2018. *JAMA Netw Open*. Published June 17, 2024.
doi:10.1001/jamanetworkopen.2024.15445

Data
Data available: No

Additional Information
Explanation for why data not available: Several data sources are proprietary and covered by data use agreements in place with third-party providers.
